# Fast DNA Serotyping and Antimicrobial Resistance Gene Determination of *Salmonella enterica* with an Oligonucleotide Microarray-Based Assay

**DOI:** 10.1371/journal.pone.0046489

**Published:** 2012-10-04

**Authors:** Sascha D. Braun, Albrecht Ziegler, Ulrich Methner, Peter Slickers, Silke Keiling, Stefan Monecke, Ralf Ehricht

**Affiliations:** 1 Alere Technologies GmbH, Jena, Germany; 2 Institute of Bacterial Infections and Zoonoses at the Friedrich-Loeffler-Institute, Federal Research Institute for Animal Health, Jena, Germany; 3 Institute for Medical Microbiology and Hygiene, Technical University of Dresden, Dresden, Germany; Institut National de la Recherche Agronomique, France

## Abstract

Salmonellosis caused by *Salmonella* (*S.*) belongs to the most prevalent food-borne zoonotic diseases throughout the world. Therefore, serotype identification for all culture-confirmed cases of *Salmonella* infection is important for epidemiological purposes. As a standard, the traditional culture method (ISO 6579:2002) is used to identify *Salmonella*. Classical serotyping takes 4–5 days to be completed, it is labor-intensive, expensive and more than 250 non-standardized sera are necessary to characterize more than 2,500 *Salmonella* serovars currently known. These technical difficulties could be overcome with modern molecular methods. We developed a microarray based serogenotyping assay for the most prevalent *Salmonella* serovars in Europe and North America. The current assay version could theoretically discriminate 28 O-antigens and 86 H-antigens. Additionally, we included 77 targets analyzing antimicrobial resistance genes. The *Salmonella* assay was evaluated with a set of 168 reference strains representing 132 serovars previously serotyped by conventional agglutination through various reference centers. 117 of 132 (81%) tested serovars showed an unique microarray pattern. 15 of 132 serovars generated a pattern which was shared by multiple serovars (*e.g.*, *S.* ser. Enteritidis and *S.* ser. Nitra). These shared patterns mainly resulted from the high similarity of the genotypes of serogroup A and D1. Using patterns of the known reference strains, a database was build which represents the basis of a new PatternMatch software that can serotype unknown *Salmonella* isolates automatically. After assay verification, the *Salmonella* serogenotyping assay was used to identify a field panel of 105 *Salmonella* isolates. All were identified as *Salmonella* and 93 of 105 isolates (88.6%) were typed in full concordance with conventional serotyping. This microarray based assay is a powerful tool for serogenotyping.

## Introduction

Salmonellosis caused by salmonellae belongs to the most prevalent food-borne zoonotic diseases throughout the world [1]. Therefore, serotype identification for all culture-confirmed cases of *Salmonella* infection is important for epidemiological purposes. The genus *Salmonella* includes two species: *Salmonella* (*S.*) *enterica* and *Salmonella bongori*. The species *Salmonella enterica* is divided into the following six subspecies: *S. enterica* subsp. *enterica* (I), *S. enterica* subsp. *salamae* (II), *S. enterica* subsp. *arizonae* (IIIa), *S. enterica* subsp. *diarizonae* (IIIb), *S. enterica* subsp. *houtenae* (IV) and *S. enterica* subsp. *indica* (VI) [2]. The subspecies *Salmonella enterica* subsp. *enterica* (I) includes the most relevant zoonotic pathogens with a global occurrence. A serotyping scheme, proposed by Kauffmann 1934 [3], divides all subspecies into serovars by immunologic analyses of two surface structures, O-polysacharide (O-antigen) and flagellin protein (H-antigen). The Kauffman-White scheme was expanded from 44 serovars in 1934 to 2,587 serovars currently known [2].

Genes required for the biosynthesis of the O-antigen are organized in the *rfb* cluster [4], [5]. Within this cluster, sequences of the sugar transferases are relatively conserved and two genes are responsible for most of the genotypic and phenotypic differences of the 46 *Salmonella* O-serogroups described in the Kauffmann-White scheme. The genes of the O-antigen flippase (*wzx*) and polymerase (*wzy*) are highly variable and specific for their respective serogroup [5], [6]. The H-antigen used for serotyping is encoded by two flagellar structure genes; *fliC* (phase 1 flagellin) and *fljB* (phase 2 flagellin). Both genes are highly conserved at their 5′ and 3′ ends and variable in their central region [7], [8]. Most *Salmonella* serovars are diphasic where *fliC* or *fljB* is expressed alternately. Serovars with only one H-phase are considered to be monophasic. Monophasic *Salmonella* could theoretically originate in two different ways. They either might represent ancestral forms which lack the second flagellar antigen and did not yet evolve the necessary switching mechanism. Alternatively, they could be deletion mutants of biphasic salmonellae that have lost either the switching mechanism or the ability to express the second flagellar antigen [9]. The genetic switching between these two phases is regulated by the *hin* gene, coding for a DNA-invertase [10]. This approximately 900-base pair (bp) DNA fragment adjacent to *fljB*, which specifies the synthesis of the H2 flagellar antigen, can exist in either orientation with respect to *fljB*. The orientation of the inversion region controls the expression of *fljB*, *i.e*., in one orientation the adjacent *fljB* is expressed and in the opposite orientation *fljB* is not expressed [11].

As a gold standard, the traditional culture method is used to detect *Salmonella* and, since 2002, ISO 6579 represents a legislative norm for the detection of *Salmonella* [12]. This method includes the non-selective pre-enrichment in buffered peptone water followed by selective enrichment and plating on two solid selective media. Colonies of interest are confirmed biochemically and serologically by agglutination with specific sera. However, the procedure according to ISO 6579:2002 takes 4–5 days to be completed. Additionally, classical serotyping is labor-intensive, expensive, requires highly experienced laboratory staff and more than 250 reagents [13] that are necessary to characterize more than 2,500 *Salmonella* serovars currently listed. Besides, commercially available sera are not standardized and their availability is often limited due to a lack of resources and funding. In contrast, genotyping methods use DNA sequence information for identification. Such sequence information is unique and techniques can easily be reproduced and standardized between different laboratories. For this reason, there is an increasing need for a simple genotyping method that does not require a stock of different sera, but can be performed automatically in high throughput and for which reagents are available worldwide. Different molecular typing systems have been developed to meet this demand, such as multiplex real time PCR [14], [15], primer extension [16], microarrays [13], [17], DNA sequence approaches [18], bead-based suspension arrays [19], [20] and ligation based microarrays [21]. Some recent molecular techniques have the disadvantage that only a small subset of serotypes can be typed whereas other approaches do not provide an antigenic formula compatible with the Kauffmann-White scheme. Some techniques are too expensive and/or labor intensive to be implemented in public health or diagnostic laboratories.

Ballmer et al (2007) proposed a genotyping microarray for *Escherichia* (*E.*) *coli* [6]. Using a comparable system we aim to develop a high throughput, economical, array-based system to serotype *Salmonella* via its genotype. The microarray includes 255 different targets to analyze O- and H-phases and assign the genotype to the antigenic formula according to the Kauffmann-White scheme. Additionally, we included 77 targets related to antimicrobial resistance (AMR). Validation and testing of the array was completed with 132 different *Salmonella* serovars, including the most prevalent *Salmonella* serovars from human and non-human sources from North America and Europe ([1], www.cdc.gov/ncezid/dfwed/PDFs/SalmonellaAnnualSummaryTables2009.pdf) to ensure the development of a comprehensive assay with a global scope.

## Methods

### Bacterial strains, growth conditions, and genomic DNA extraction

The microarray based assay was evaluated with a set of 168 reference strains representing 132 serovars previously serotyped by conventional agglutination through various reference centers, including the Centers for Disease Control and Prevention (CDC, Atlanta, USA), German Collection of Microorganism and Cell Cultures (DSMZ, Brunswick, Germany), Salmonella Genetic Stock Center (SGSC, Calgary, Canada) and National Reference Laboratory for Salmonellosis in Cattle at the Friedrich-Loeffler-Institute (FLI, Jena, Germany) (Table 1). For the *S.* serovar (ser.) Typhimurium strain LT2, the complete genome sequence (GenBank NC_003197.1), the antigenic formula and a theoretical prediction of the microarray hybridization pattern (which was generated using a probe-matching matrix; see Table S1), were available. The strains were cultivated on tryptone yeast agar, and genomic DNA was extracted using a Roche High Pure PCR Template Preparation Kit (Roche Diagnostics, Germany) or a Qiagen DNeasy Blood & Tissue Kit (Qiagen GmbH, Hilden, Germany) according to manufacturer's instructions after treatment with lysis enhancer (Alere Technologies, Germany). If necessary, DNA was concentrated to at least 100 ng/µl using a SpeedVac centrifuge (Eppendorf, Hamburg, Germany) at room temperature for 30 min/1,400 rpm.

**Table 1:** *Salmonella* strains used to validate the *Salmonella* serogenotyping array.

Species	Serovar	Strain	Results of classical Serotyping		Results of microarray based Serotyping			
			Serogroup	Antigenic Formula	Serogroup	*invA*/*galF*/*manC* a	Unique Pattern	Pattern similar to Serovars
*S.e. enterica*	Paratyphi A	CDC1	A (O:2)	1,2,12:a:[1,5]	A (O:2)	+/+/+	Yes	
*S.e. enterica*	Nitra	CDC1280	A (O:2)	2,12:g,m:-	A (O:2)	+/+/+	No	Enteritidis, Blegdam
*S.e. enterica*	Kiel	CDC09-1879; CDC674	A (O:2)	1,2,12:g,p:-	A (O:2)	+/+/+	No	Dublin, Naestved, Moscow
*S.e. enterica*	Koessen	CDC2417	A (O:2)	2,12:l,v:1,5	A (O:2)	+/+/+	No	Panama
*S.e. enterica*	Abony	CDC103; DSM4224	B (O:4)	1,4,[5],12,[27]:b:e,n,x	B (O:4)	+/+/+	Yes	
*S.e. enterica*	Paratyphi B	CDC3	B (O:4)	1,4,[5],12:b:1,2	B (O:4)	+/+/+	Yes	
*S.e. enterica*	Wien	SGSC2528	B (O:4)	1,4,12,[27]:b:l,w	B (O:4)	+/+/+	Yes	
*S.e. enterica*	Jericho	CDC621	B (O:4)	1,4,12,27:c:e,n,z15	B (O:4)	+/+/+	Yes	
*S.e. enterica*	Duisburg	SGSC2472	B (O:4)	1,4,12,[27]:d:e,n,z15	B (O:4)	+/+/+	Yes	
*S.e. enterica*	Schwarzengrund	CDC1629; SGSC2514	B (O:4)	1,4,12,27:d:1,7	B (O:4)	+/+/+	Yes	
*S.e. enterica*	Stanley	CDC000477; SGSC2517	B (O:4)	1,4,[5],12,[27]:d:1,2	B (O:4)	+/+/+	Yes	
*S.e. enterica*	Chester	CDC17	B (O:4)	1,4,[5],12:e,h:e,n,x	B (O:4)	+/+/+	Yes	
*S.e. enterica*	Reading	CDC19; SGSC2510	B (O:4)	1,4,[5],12:e,h:1,5	B (O:4)	+/+/+	Yes	
*S.e. enterica*	Saintpaul	CDC108	B (O:4)	1,4,[5],12:e,h:1,2	B (O:4)	+/+/+	Yes	
*S.e. enterica*	Sandiego	CDC18	B (O:4)	1,4,[5],12:e,h:e,n,z15	B (O:4)	+/+/+	Yes	
*S.e. enterica*	Derby	CDC20	B (O:4)	1,4,[5],12:f,g:[1,2]	B (O:4)	+/+/+	Yes	
*S.e. enterica*	Agona	CDC1636	B (O:4)	1,4,[5],12:f,g,s:[1,2]	B (O:4)	+/+/+	Yes	
*S.e. enterica*	California	CDC1109	B (O:4)	4,12:g,m,t:[z67]	B (O:4)	+/+/+	Yes	
*S.e. enterica*	Budapest	CDC23	B (O:4)	1,4,12,[27]:g,t:-	B (O:4)	+/+/+	Yes	
*S.e. enterica*	Travis	CDC990318	B (O:4)	4,[5],12:g,z51:1,7	B (O:4)	+/+/+	Yes	
*S.e. enterica*	1,4,[5],12:i:-	CDCQA126; NRL688; NRL813	B (O:4)	1,4,[5],12:i:-	B (O:4)	+/+/+	Yes	
*S.e. enterica*	Agama	CDC513	B (O:4)	4,12:i:1,6	B (O:4)	+/+/+	Yes	
*S.e. enterica*	Gloucester	CDC443	B (O:4)	1,4,12,27:i:l,w	B (O:4)	+/+/+	Yes	
*S.e. enterica*	Typhimurium	CDC14; DSM10506; DSM17058; DSM17058; DSM19587; DSM554; LT2	B (O:4)	1,4,[5],12:i:1,2	B (O:4)	+/+/+	Yes	
*S.e. enterica*	Brandenburg	CDC2519; SGSC2460	B (O:4)	4,[5],12:l,v:e,n,z15	B (O:4)	+/+/+	Yes	
*S.e. enterica*	Bredeney	CDC112	B (O:4)	1,4,12,27:l,v:1,7	B (O:4)	+/+/+	Yes	
*S.e. enterica*	Heidelberg	CDC16; DSM9379	B (O:4)	1,4,[5],12:r:1,2	B (O:4)	+/+/+	Yes	
*S.e. enterica*	Indiana	CDC377; SGSC2482	B (O:4)	1,4,12:z:1,7	B (O:4)	+/+/+	No	Kiambu
*S.e. enterica*	Kiambu	CDC399	B (O:4)	1,4,12:z:1,5	B (O:4)	+/+/+	No	Indiana
*S.e. enterica*	Haifa	SGSC2479	B (O:4)	1,4,[5],12:z10:1,2	B (O:4)	+/+/+	Yes	
*S.e. enterica*	Stanleyville	CDC223; SGSC2518	B (O:4)	1,4,[5],12,[27]:z4,z23:[1,2]	B (O:4)	+/+/+	Yes	
*S.e. enterica*	Maska	CDC2349	B (O:4)	1,4,12,27:z41:e,n,z15	B (O:4)	+/+/+	Yes	
*S.e. enterica*	Ohio	CDC710	C1 (O:7)	6,7,14:b:l,w	C1 (O:7)	+/+/+	Yes	
*S.e. enterica*	Choleraesuis	CDC34; DSM14846	C1 (O:7)	6,7:c:1,5	C1 (O:7)	+/+/+	Yes	
*S.e. enterica*	Paratyphi C	CDC33; SGSC3592	C1 (O:7)	6,7,[Vi]:c:1,5	C1 (O:7)	+/+/+	Yes	
*S.e. enterica*	Typhisuis	SGSC2527	C1 (O:7)	6,7:c:1,5	C1 (O:7)	+/+/+	Yes	
*S.e. enterica*	Kambole	CDC1863	C1 (O:7)	6,7:d:1,[2],7	C1 (O:7)	+/+/+	Yes	
*S.e. enterica*	Livingstone	NRL720	C1 (O:7)	6,7,14:d:l,w	C1 (O:7)	+/+/+	Yes	
*S.e. enterica*	Braenderup	CDC49	C1 (O:7)	6,7,14:e,h:e,n,z15	C1 (O:7)	+/+/+	Yes	
*S.e. enterica*	Nola	CDC2206	C1 (O:7)	6,7:e,h:1,7	C1 (O:7)	+/+/+	Yes	
*S.e. enterica*	Rissen	CDC955	C1 (O:7)	6,7,14:f,g:-	C1 (O:7)	+/+/+	Yes	
*S.e. enterica*	Montevideo	CDC1904	C1 (O:7)	6,7,14:g,m,[p],s:[1,2,7]	C1 (O:7)	+/+/+	Yes	
*S.e. enterica*	Singapore	CDC010011	C1 (O:7)	6,7:k:e,n,x	C1 (O:7)	+/+/+	Yes	
*S.e. enterica*	Thompson	CDC000342	C1 (O:7)	6,7,14:k:1,5	C1 (O:7)	+/+/+	Yes	
*S.e.diarizonae*	6,7:l,v:z53	DSM14847	C1 (O:7)	6,7:l,v:z53	C1 (O:7)	+/+/+	Yes	
*S.e. enterica*	Bonn	CDC344	C1 (O:7)	6,7:l,v:e,n,x	C1 (O:7)	+/+/+	Yes	
*S.e. enterica*	Potsdam	CDC876	C1 (O:7)	6,7,14:l,v:e,n,z15	C1 (O:7)	+/+/+	Yes	
*S.e. enterica*	Kenya	CDC497	C1 (O:7)	6,7:l,z13:e,n,x	C1 (O:7)	+/+/+	Yes	
*S.e. enterica*	Haelsingborg	CDC586	C1 (O:7)	6,7:m,p,t,[u]:-	C1 (O:7)	+/+/+	Yes	
*S.e. enterica*	Oranienburg	CDC1271	C1 (O:7)	6,7,14:m,t:[z57]	C1 (O:7)	+/+/+	Yes	
*S.e. enterica*	Infantis	CDC1428	C1 (O:7)	6,7,14:r:1,5	C1 (O:7)	+/+/+	Yes	
*S.e. enterica*	Virchow	CDC2688	C1 (O:7)	6,7,14:r:1,2	C1 (O:7)	+/+/+	Yes	
*S.e. enterica*	Bareilly	NRL608	C1 (O:7)	6,7,14:y:1,5	C1 (O:7)	+/+/+	Yes	
*S.e. enterica*	Mbandaka	CDC1906	C1 (O:7)	6,7,14:z10:e,n,z15	C1 (O:7)	+/+/+	Yes	
*S.e. enterica*	Tennessee	CDC155	C1 (O:7)	6,7,14:z29:[1,2,7]	C1 (O:7)	+/+/+	Yes	
*S.e. enterica*	Tienba	CDC2425	C1 (O:7)	6,7:z35:1,6	C1 (O:7)	+/+/+	Yes	
*S.e. enterica*	Lille	CDC354	C1 (O:7)	6,7,14:z38:-	C1 (O:7)	+/+/+	Yes	
*S.e. enterica*	Manhattan	CDC122	C2–C3 (O:8)	6,8:d:1,5	C2–C3 (O:8)	+/+/+	Yes	
*S.e. enterica*	Muenchen	CDC54; SGSC2243	C2–C3 (O:8)	6,8:d:1,2	C2–C3 (O:8)	+/+/+	Yes	
*S.e. enterica*	Virginia	CDC189	C2–C3 (O:8)	8:d:1,2	C2–C3 (O:8)	+/+/+	Yes	
*S.e. enterica*	Kottbus	CDC52	C2–C3 (O:8)	6,8:e,h:1,5	C2–C3 (O:8)	+/+/+	Yes	
*S.e. enterica*	Newport	CDC2434	C2–C3 (O:8)	6,8,20:e,h:1,2	C2–C3 (O:8)	+/+/+	Yes	
*S.e. enterica*	Emek	SGSC2477	C2–C3 (O:8)	8,20:g,m,s:-	C2–C3 (O:8)	+/+/+	Yes	
*S.e. enterica*	Kentucky	CDC2590; Eng196^b^	C2–C3 (O:8)	8,20:i:z6	C2–C3 (O:8)	+/+/+	Yes	
*S.e. enterica*	Lindenburg	CDC334	C2–C3 (O:8)	6,8:i:1,2	C2–C3 (O:8)	+/+/+	Yes	
*S.e. enterica*	Blockley	CDC448; Eng23^b^; Eng24^b^	C2–C3 (O:8)	6,8:k:1,5	C2–C3 (O:8)	+/+/+	Yes	
*S.e. enterica*	Litchfield	CDC000462	C2–C3 (O:8)	6,8:l,v:1,2	C2–C3 (O:8)	+/+/+	Yes	
*S.e. enterica*	Manchester	Eng205^b^	C2–C3 (O:8)	6,8:l,v:1,7	C2–C3 (O:8)	+/+/+	Yes	
*S.e. enterica*	Breukelen	CDC1699	C2–C3 (O:8)	6,8:l,z13,[z28]:e,n,z15	C2–C3 (O:8)	+/+/+	Yes	
*S.e. enterica*	Goldcoast	NRL852	C2–C3 (O:8)	6,8:r:l,w	C2–C3 (O:8)	+/+/+	Yes	
*S.e. enterica*	Bovismorbificans	CDC2201	C2–C3 (O:8)	6,8,20:r,[i]:1,5	C2–C3 (O:8)	+/+/+	Yes	
*S.e. enterica*	Hidalgo	CDC2359	C2–C3 (O:8)	6,8:r,[i]:e,n,z15	C2–C3 (O:8)	+/+/+	Yes	
*S.e. enterica*	Hadar	CDC347;	C2–C3 (O:8)	6,8:z10:e,n,x	C2–C3 (O:8)	+/+/+	No	Istanbul
*S.e. enterica*	Istanbul	CDC1466	C2–C3 (O:8)	8:z10:e,n,x	C2–C3 (O:8)	+/+/+	No	Hadar
*S.e. enterica*	Uno	CDC1697	C2–C3 (O:8)	6,8:z29:[e,n,z15]	C2–C3 (O:8)	+/+/+	Yes	
*S.e. enterica*	Corvallis	CDC1770	C2–C3 (O:8)	8,20:z4,z23:[z6]	C2–C3 (O:8)	+/+/+	Yes	
*S.e. enterica*	Duesseldorf	CDC130	C2–C3 (O:8)	6,8:z4,z24:-	C2–C3 (O:8)	+/+/+	Yes	
*S.e. enterica*	Tallahassee	CDC196	C2–C3 (O:8)	6,8:z4,z32:-	C2–C3 (O:8)	+/+/+	Yes	
*S.e. enterica*	Gallinarum	CDC74; DSM13674	D1 (O:9)	1,9,12:-:-	D1 (O:9)	+/+/+	Yes	
*S.e. enterica*	Berta	CDC69	D1 (O:9)	1,9,12:[f],g,[t]:-	D1 (O:9)	+/+/+	Yes	
*S.e. enterica*	Miami	CDC198; SGSC2485	D1 (O:9)	1,9,12:a:1,5	D1 (O:9)	+/+/+	Yes	
*S.e. enterica*	Goeteborg	CDC696	D1 (O:9)	9,12:c:1,5	D1 (O:9)	+/+/+	Yes	
*S.e. enterica*	Typhi	No. 1^c^	D1 (O:9)	9,12[Vi]:d:-	D1 (O:9)	+/+/+	Yes	
*S.e. enterica*	Enteritidis	CDC64; DSM14221; DSM17420	D1 (O:9)	1,9,12:g,m:-	D1 (O:9)	+/+/+	No	Nitra, Blegdam
*S.e. enterica*	Blegdam	CDC090361; CDC68	D1 (O:9)	9,12:g,m,q:-	D1 (O:9)	+/+/+	No	Nitra, Enteritidis
*S.e. enterica*	Dublin	CDC10-0635; CDC65	D1 (O:9)	1,9,12[Vi]:g,p:-	D1 (O:9)	+/+/+	No	Kiel, Naestved, Moscow
*S.e. enterica*	Naestved	CDC559; SGSC3612	D1 (O:9)	1,9,12:g,p,s:-	D1 (O:9)	+/+/+	No	Kiel, Dublin, Moscow
*S.e. enterica*	Moscow	CDC67	D1 (O:9)	1,9,12:g,q:-	D1 (O:9)	+/+/+	No	Kiel, Dublin, Naestved
*S.e. enterica*	Panama	CDC73; SGSC2496	D1 (O:9)	1,9,12:l,v:1,5	D1 (O:9)	+/+/+	No	Koessen
*S.e. salamae*	9:l,w:e,n,x	DSM9220	D1 (O:9)	9:l,w:e,n,x	D1 (O:9)	+/+/+	Yes	
*S.e. enterica*	Javiana	CDC146	D1 (O:9)	1,9,12:l,z28:1,5	D1 (O:9)	+/+/+	Yes	
*S.e. enterica*	Ottawa	CDC1934	D1 (O:9)	1,9,12:z41:1,5	D1 (O:9)	+/+/+	Yes	
*S.e. enterica*	Franken	CDC2570	D1 (O:9)	9,12:z6:z67	D1 (O:9)	+/+/+	Yes	
*S.e. enterica*	Fresno	CDC1412	D2 (O:9,46)	9,46:z38:-	D2 (O:9,46)	+/+/+	Yes	
*S.e. enterica*	Anatum	CDC78	E1 (O:3,10)	3,{10}{15}{15,34}:e,h:1,6	E1 (O:3,10)	+/+/+	Yes	
*S.e. enterica*	Meleagridis	NRL737	E1 (O:3,10)	3,{10}{15}{15,34}:e,h:l,w	E1 (O:3,10)	+/+/+	Yes	
*S.e. enterica*	Muenster	CDC79	E1 (O:3,10)	3,{10}{15}{15,34}:e,h:1,5	E1 (O:3,10)	+/+/+	Yes	
*S.e. enterica*	Amsterdam	CDC070756	E1 (O:3,10)	3,{10}{15}{15,34}:g,m,s:-	E1 (O:3,10)	+/+/+	Yes	
*S.e. enterica*	Westhampton	CDC326	E1 (O:3,10)	3,{10}{15}{15,34}:g,s,t:-	E1 (O:3,10)	+/+/+	No	Senftenberg
*S.e. enterica*	Bessi	CDC1999	E1 (O:3,10)	3,10:i:e,n,x	E1 (O:3,10)	+/+/+	Yes	
*S.e. enterica*	Give	CDC495; CDC77	E1 (O:3,10)	3,{10}{15}{15,34}:l,v:1,7	E1 (O:3,10)	+/+/+	Yes	
*S.e. enterica*	London	NRL700	E1 (O:3,10)	3,{10}{15}:l,v:1,6	E1 (O:3,10)	+/+/+	Yes	
*S.e. enterica*	Weltevreden	CDC147	E1 (O:3,10)	3,{10}{15}:r:z6	E1 (O:3,10)	+/+/+	Yes	
*S.e. enterica*	Orion	CDC321	E1 (O:3,10)	3,{10}{15}{15,34}:y:1,5	E1 (O:3,10)	+/+/+	Yes	
*S.e. enterica*	Pietersburg	CDC2258	E1 (O:3,10)	3,{10}{15,34}:z69:1,7	E1 (O:3,10)	+/+/+	Yes	
*S.e. enterica*	Senftenberg	CDC87; DSM10062	E4 (O:1,3,19)	1,3,19:g,[s],t:-	E4 (O:1,3,19)	+/+/+	No	Westhampton
*S.e. enterica*	Westerstede	CDC607	E4 (O:1,3,19)	1,3,19:l,z13:1,2	E4 (O:1,3,19)	+/+/+	Yes	
*S.e. enterica*	Missouri	CDC2309	F (O:11)	11:g,s,t:-	F (O:11)	+/+/+	Yes	
*S.e. enterica*	Connecticut	CDC2392	F (O:11)	11:l,z13,z28:1,5	F (O:11)	+/+/+	Yes	
*S.e. enterica*	Rubislaw	CDC102; SGSC2511	F (O:11)	11:r:e,n,x	F (O:11)	+/+/+	Yes	
*S.e. enterica*	Mississippi	CDC154	G (O:13)	1,13,23:b:1,5	G (O:13)	+/+/+	Yes	
*S.e. enterica*	Havana	NRL607	G (O:13)	1,13,23:f,g,[s]:-	G (O:13)	+/+/+	Yes	
*S.e. enterica*	Idikan	CDC1690	G (O:13)	1,13,23:i:1,5	G (O:13)	+/+/+	Yes	
*S.e. enterica*	Kedougou	CDC1523	G (O:13)	1,13,23:i:l,w	G (O:13)	+/+/+	Yes	
*S.e. enterica*	Poona	CDC1243	G (O:13)	1,13,22:z:1,6	G (O:13)	+/+/+	Yes	
*S.e. enterica*	Cubana	CDC207	G (O:13)	1,13,23:z29:-	G (O:13)	+/+/+	Yes	
*S.e. enterica*	Ajiobo	CDC527	G (O:13)	13,23:z4,z23:-	G (O:13)	+/+/+	Yes	
*S.e. indica*	6,14:a:e,n,x	DSM14848	G (O:13)	6,14:a:e,n,x	G (O:13)	+/+/+	Yes	
*S.e. enterica*	Blijdorp	CDC765	H (O:6,14)	1,6,14,25:c:1,5	H (O:6,14)	+/+/+	Yes	
*S.e. enterica*	Carrau	CDC93	H (O:6,14)	6,14,[24]:y:1,7	H (O:6,14)	+/+/+	Yes	
*S.e. enterica*	Grancanaria	CDC2506	I (O:16)	16:z39:[1,6]	I (O:16)	+/+/+	Yes	
*S.e. enterica*	Cerro	CDC990087	K (O:18)	6,14,18:z4,z23:[1,5]	K (O:18)	+/+/+	Yes	
*S.e. enterica*	Pomona	CDC2473A	M (O:28)	28:y:1,7	M (O:28)	+/+/+	Yes	
*S.e. enterica*	Morocco	CDC694	N (O:30)	30:l,z13,z28:e,n,z15	N (O:30)	+/+/+	Yes	
*S.e. enterica*	Ealing	CDC745	O (O:35)	35:g,m,s:-	O (O:35)	+/+/+	Yes	
*S.e. enterica*	Alachua	CDC325	O (O:35)	35:z4,z23:-	O (O:35)	+/+/+	Yes	
*S.e. enterica*	Kasenyi	NRL878	P (O:38)	38:e,h:1,5	P (O:38)	+/+/+	Yes	
*S.e. enterica*	Lansing	CDC634	P (O:38)	38:i:1,5	P (O:38)	+/+/+	Yes	
*S.e. enterica*	Inverness	CDC171	P (O:38)	38:k:1,6	P (O:38)	+/+/+	Yes	
*S.e. enterica*	Gera	CDC1316	T (O:42)	1,42:z4,z23:1,6	T (O:42)	+/+/+	Yes	
*S.e. enterica*	Niederoderwitz	CDC2579	U (O:43)	43:b:-	U (O:43)	+/+/+	Yes	
*S. bongori*	66:z41:-	DSM13774	O:66	66:z41:-	O:66	+/+/+	Yes	

a *invA*, *galF* and *manC* are species marker for *Salmonella*.

b generous gift of Paul Barrow, University of Nottingham Sutton Bonington Campus, UK.

c only genomic DNA of *Salmonella* Typhi, generous gift of Rene S. Hendrickson, DTU Food, Denmark.

Strains were classically serotyped by the CDC (Centers of Disease Control and Prevention, Atlanta, USA), DSMZ (German Collection of Microorganism and Cell Cultures, Brunswick, Germany), SGSC (Salmonella Genetic Stock Center, Calgary, Canada) and FLI (National Reference Laboratory for Salmonellosis in cattle at the Friedrich-Loeffler-Institute, Jena, Germany).

### Array design

Discrimination of the 46 described O-serotypes is mainly determined by the genes *wzy* (polymerase) and *wzx* (flippase). The 114 known H-antigens are encoded by two genes; *fliC* (phase 1 flagellin) and *fljB* (phase 2 flagellin). The resulting 24- to 34-bp primer and oligonucleotide probes for serogenotyping were selected from variable parts of multiple sequence alignments for these determinative genes aiming to be as discriminating as possible and to contain similar G+C contents to ensure a very similar hybridization behavior. 255 serotyping probes were designed by analyzing all available annotated GenBank sequences (NCBI, http://www.ncbi.nlm.nih.gov/) related to the genes *wzx* and *wzy* as well as *fliC* and *fljB*. Additionally, the genes *manC* (O7, O11, O18, O40, O41), *wbuH* (O41, O62), *weiB* (O66), and *rfbV* (O4) were used to discriminate O-serotypes (Table 2). The probes immobilized on the current array version can discriminate 28 O-antigens: A (O:2), B (O:4), C1 (O:7), C2–C3 (O:8), D1 (O:9), D2 (O:9,46), E1/E4 (O:3,10/O:1,3,19), F (O:11), G (O:13), H (O:6,14), I (O:16), J (O:17), K (O:18), M (O:28), N (O:30), O (O:35), P (O:38), Q (O:39), R (O:40), S (O:41), U (O:43), W (O:45), Z (O:50), O55, O56, O58, O62 and O66. From this set, 19 serogroups (A, B, C1, C2–C3, D1, D2, E1, E4, F, G, H, I, K, M, N, O, P, T, U) were tested on the microarray using the 132 reference serovars (Table 1). Due to the high similarity between the serogroups A and D1, additional probes were designed to discriminate *S.* ser. Nitra and *S*. ser. Enteritidis. For this purpose, specific probes located in the genes *lygA*, *lygD*, *sefA*, *sefB* and *sefC* were designed to specifically identify *S.* ser. Enteritidis (Table 2). In order to identify *S.* ser. Paratyphi A, probes were designed to target the intergenic region SSPAI, a genomic island next to *clpA* [22]. For the discrimination of *S*. ser. Dublin from *S*. ser. Kiel, the genes *SeD_A1100*, *SeD_A1101* and *SeD_A1102* were used as they code for a conserved putative protein being specific for serovar Dublin [23].

**Table 2:** Summary of the potential function(s) on the basis of classical serotyping of each probe immobilized on the microarray.

Probe	Potential Function	Probe	Potential Function	Probe	Potential function
hp-3001-FL-1+e,n,x	e,n,x; e,n,x,z15; z6	hp-3117-FL-l+z39+z52	z39	hp-3219-wzx_O35	O35
hp-3003-FL-1+e,n,x	1,5; 1,6; e,n,x; e,n,x,z15; z6	hp-3118-FL-l+z39+z52	z52	hp-3220-wzx_O4	O4
hp-3004-FL-1+e,n,x	1,11,16; 1,12; 1,2; 1,2,7; 1,5; 1,5,7; 1,6; 1,7; z	hp-3120-FL-g,z51	g,z51	hp-3221-wzx_O4	O4
hp-3005-FL-1+e,n,x	1,11,16; 1,12; 1,2; 1,2,7; 1,5; 1,5,7; 1,6; 1,7; z	hp-3121-FL-g,z51	g,z51	hp-3222-wzx_O4	O4
hp-3006-FL-1+e,n,x	1,11,16; 1,12; 1,2; 1,2,7; 1,5; 1,5,7; 1,6; 1,7; z	hp-3124-FL-e,n,x	e,n,x; e,n,z15	hp-3223-wzx_O41+62	O41; O62
hp-3007-FL-1+e,n,x	1,5; 1,6	hp-3125-FL-b+z91	b; z91	hp-3224-wzx_O41+62	O41; O62
hp-3008-FL-1+e,n,x	1,2; 1,5; 1,6; e,n,x; e,n,x,z15; z6	hp-3126-FL-b+z91	b; z91	hp-3225-wzx_O50	O50
hp-3009-FL-1+e,n,x	1,2; 1,5; 1,6; e,n,x; e,n,x,z15; z6	hp-3128-FL-b+z91	b; z91	hp-3226-wzx_O50	O50
hp-3012-FL-1+e,n,x	1,11,16; 1,12; 1,2; 1,2,7; 1,5; 1,5,7; 1,6; 1,7; e,n,x; e,n,x,z15; z; z6	hp-3129-FL-b+z91	b; z91	hp-3227-wzx_O55	O55
hp-3013-FL-1+e,n,x	1,11,16; 1,12; 1,2; 1,2,7; 1,5; 1,5,7; 1,6; 1,7; e,n,x; e,n,x,z15; z; z6	hp-3130-FL-b+z91	b; z91	hp-3228-wzx_O55	O55
hp-3014-FL-1+e,n,x	1,11,16; 1,12; 1,2; 1,2,7; 1,5; 1,5,7; 1,6; 1,7; e,n,x; e,n,x,z15; z; z6	hp-3134-FL-z	z6	hp-3229-wzx_O56	O56
hp-3015-FL-1+e,n,x	1,11,16; 1,12; 1,2; 1,2,7; 1,5; 1,6; 1,7; e,n,x; e,n,x,z15; z; z6	hp-3135-FL-z	z6	hp-3230-wzx_O56	O56
hp-3016-FL-c	c	hp-3136-FL-z	z69	hp-3231-wzx_O58	O58
hp-3017-FL-c	c	hp-3138-FL-z	z	hp-3232-wzx_O58	O58
hp-3018-FL-d+j	d	hp-3139-FL-z	z	hp-3233-wzx_O6,14	O6,14
hp-3019-FL-d+j	d	hp-3140-FL-z	z	hp-3234-wzx_O6,14	O6,14
hp-3020-FL-d+j	d	hp-3141-FL-z	z50	hp-3235-wzx_O66	O66
hp-3021-FL-d+j	d; j	hp-3142-FL-z	z; z35	hp-3236-wzx_O66	O66
hp-3022-FL-d+j	d	hp-3144-FL-z	z50	hp-3237-wzx_O7	O7
hp-3023-FL-d+j	d; j	hp-3145-FL-z	z	hp-3238-wzx_O7	O7
hp-3024-FL-e,h	e,h	hp-3146-FL-z	z	hp-3239-wzx_O7	O7
hp-3025-FL-e,h	e,h	hp-3149-FL-l+z39+z52	z39	hp-3240-wzx_O8	O8
hp-3026-FL-e,n,x	e,n,x,z15	hp-3150-FL-z	z	hp-3241-wzx_O8	O8
hp-3027-FL-e,n,x	e,n,x; e,n,x,z15	hp-3152-FL-i+r	i	hp-3242-wzy_O13	O13
hp-3029-FL-g,z51	g,z51	hp-3153-FL-l+z39+z52	l,v; l,w; l,z13; l,z13,z28; l,z28	hp-3243-wzy_O13	O13
hp-3032-FL-i+r	i	hp-3154-FL-k+z	z10	hp-3244-wzy_O16	O16
hp-3033-FL-i+r	i	hp-3155-FL-z4	z4,z23; z4,z24; z4,z32	hp-3245-wzy_O16	O16
hp-3034-FL-i+r	i	hp-3157-FL-1+e,n,x	1,11,16; 1,12; 1,2; 1,2,7; 1,5; 1,5,7; 1,6; 1,7; e,n,x; e,n,x,z15; z; z6	hp-3246-wzy_O17	O17
hp-3035-FL-i+r	r	hp-3158-FL-1+e,n,x	1,11,16; 1,12; 1,2; 1,2,7; 1,5; 1,5,7; 1,6; 1,7; e,n,x; e,n,x,z15; z; z6	hp-3247-wzy_O17	O17
hp-3036-FL-i+r	r	hp-3161-FL-1+e,n,x	1,5; 1,6	hp-3248-wzy_O18	O18
hp-3038-FL-k+z	k; z44; z58	hp-3163-FL-1+e,n,x	1,11,16; 1,12; 1,2; 1,2,7; 1,5; 1,5,7; 1,6; 1,7; z	hp-3250-wzy_O28_Dakar	O28 serovar Dakar
hp-3039-FL-k+z	l,v; z10; z35; z39; z65	hp-3165-manC	species marker	hp-3251-wzy_O28_Dakar	O28 serovar Dakar
hp-3040-FL-k+z	z35	hp-3166-wbyJ	O41	hp-3252-wzy_O28_Pomona	O28 serovar Pomona
hp-3041-FL-k+z	k	hp-3167-wbyJ	O41	hp-3253-wzy_O28_Pomona	O28 serovar Pomona
hp-3042-FL-k+z	k; z41	hp-3168-manC-O16+39	O16; O39	hp-3254-wzy_O3,10+9,46	O3,10; O9,46
hp-3043-FL-k+z	(k)	hp-3169-manC-O16+39	O16; O39	hp-3255-wzy_O3,10+9,46	O3,10; O9,46
hp-3044-FL-z	z41	hp-3170-manC-O7	O7	hp-3256-wzy_O3,10+9,46	O3,10; O9,46
hp-3045-FL-k+z	z10	hp-3171-manC-O7	O7	hp-3257-wzy_O30	O30
hp-3046-FL-k+z	z10	hp-3172-manC-O11	O11	hp-3258-wzy_O30	O30
hp-3047-FL-k+z	z81	hp-3173-manC-O11	O11	hp-3259-wzy_O35	O35
hp-3048-FL-k+z	a,z10	hp-3174-manC-O18	O18	hp-3260-wzy_O35	O35
hp-3049-FL-k+z	z35	hp-3175-manC-O18	O18	hp-3261-wzy_O38	O38
hp-3050-FL-k+z	k; z58; z44; z41	hp-3176-manC-O41	O41	hp-3262-wzy_O38	O38
hp-3051-FL-k+z	a; z10	hp-3177-manC-O41	O41	hp-3263-wzy_O41+62	O41; O62
hp-3052-FL-z	z41	hp-3178-manC-O41	O41	hp-3264-wzy_O41+62	O41; O62
hp-3053-FL-k+z	z81	hp-3179-manC-O13+O30+O43+O45+O50	O13; O30; O43; O45; O50	hp-3265-wzy_O50	O50
hp-3054-FL-k+z	z35	hp-3180-manC-O13+O30+O43+O45+O50	O13; O30; O43; O45; O50	hp-3266-wzy_O50	O50
hp-3055-FL-k+z	z35	hp-3181-manC-O13+O30+O43+O45+O50	O13; O30; O43; O45; O50	hp-3267-wzy_O55	O55
hp-3056-FL-k+z	z35	hp-3182-manC-O13+O30+O43+O45+O50	O13; O30; O43; O45; O50	hp-3268-wzy_O55	O55
hp-3057-FL-k+z	(k)	hp-3183-manC-O13+O30+O43+O45+O50	O13; O30; O43; O45; O50	hp-3269-wzy_O56	O56
hp-3058-FL-k+z	z10	hp-3184-manC-O13+O30+O43+O45+O50	O13; O30; O43; O45; O50	hp-3270-wzy_O56	O56
hp-3060-FL-k+z	k; z41	hp-3185-manC-O13+O30+O43+O45+O50	O13; O30; O43; O45; O50	hp-3271-wzy_O58	O58
hp-3061-FL-k+z	(k)	hp-3186-manC-O13+O30+O43+O45+O50	O13; O30; O43; O45; O50	hp-3272-wzy_O58	O58
hp-3062-FL-l+z39+z52	z39	hp-3187-manC-O13+O30+O43+O45+O50	O13; O30; O43; O45; O50	hp-3273-wzy_O6,14	O6,14
hp-3063-FL-l+z39+z52	l,v; l,z13; l,z28; l,z13,z28; l,w	hp-3188-manC-O2+4+9+3,10	O2; O4; O9; O3,10	hp-3274-wzy_O6,14	O6,14
hp-3065-FL-l+z39+z52	z52	hp-3189-manC-O2+4+9+3,10	O2; O4; O9; O3,10	hp-3275-wzy_O7	O7
hp-3066-FL-l+z39+z52	l,v; l,z13; l,z28; l,w; l,z13,z28	hp-3190-manC-O40	O40	hp-3276-wzy_O7	O7
hp-3067-FL-y	y	hp-3191-manC-O40	O40	hp-3277-wzy_O8	O8
hp-3068-FL-y	y	hp-3192-rfbV-O2+9+9,46	O2; O9; O9,46	hp-3278-wzy_O8	O8
hp-3069-FL-z29	z29	hp-3193-rfbV-O2+9+9,46	O2; O9; O9,46	hp-3279-wzy_O18	O18
hp-3070-FL-z29	z29	hp-3194-rfbV-O4	O4	hp-3280-SSPAI	Paratyphi A
hp-3071-FL-z36+z38	z38	hp-3195-rfbV-O4	O4	hp-3281-SSPAI	Paratyphi A
hp-3072-FL-z36+z38	z36; z36,z38	hp-3196-wbuH-O41+62	O41; O62	hp-3282-Q8ZK10	Typhimurum
hp-3073-FL-z36+z38	z36; z36,z38	hp-3197-wbuH-O41+62	O41; O62	hp-3287-lygA	Enteritidis
hp-3074-FL-z36+z38	z36; z36,z38	hp-3198-weiB_O66	O66	hp-3288-lygD	Enteritidis
hp-3075-FL-z36+z38	z36,z38; z38	hp-3199-weiB_O66	O66	hp-3289-Q8ZK15	Typhimurium
hp-3076-FL-z4	z4,z23; z4,z23,z32; z4,z24; z4,z32	hp-3200-wzx_O13	O13	hp-3290-tviA	plasmid Vi
hp-3077-FL-z4	z4,z24	hp-3201-wzx_O13	O13	hp-3292-tviA	plasmid Vi
hp-3078-FL-z4	z4,z23,z32	hp-3202-wzx_O16	O16	hp-3293-stgA	Typhi
hp-3080-FL-z65	z65	hp-3203-wzx_O16	O16	hp-3294-stgA	Typhi
hp-3085-FL-g	f,g; f,g,s,t; f,g,t; g,m,p,q; g,m,t; g,z62	hp-3204-wzx_O17	O17	hp-3297-sefB	Enteritidis
hp-3086-FL-g	f,g; f,g,s,t; f,g,t; g,m,p,q; g,m,s; g,m,s,t; g,m,t; g,t; g,z62	hp-3205-wzx_O17	O17	hp-3298-sefA	Enteritidis
hp-3087-FL-g	f,g; f,g,s,t; f,g,t; g,m,p,q; g,m,t; g,t; g,z62	hp-3206-wzx_O18	O18	hp-3299-sefC	Enteritidis
hp-3089-FL-g	f,g,t; g,m,t; m,t	hp-3207-wzx_O18	O18	hp-3300-galF	species marker
hp-3090-FL-g	f,g,t; g,m,t; m,t	hp-3208-wzx_O2+9	O2; O9	hp-3301-B5FQV7	Dublin
hp-3091-FL-g	f,g; f,g,s,t; f,g,t; g,m,p,q; g,m,s; g,m,s,t; g,m,t; g,t; g,z62	hp-3209-wzx_O2+9	O2; O9	hp-3302-B5R5L5	
hp-3092-FL-g	f,g; f,g,s,t; g,m,p,q; g,m,s; g,m,s,t; g,m,t; g,t; g,z62	hp-3210-wzx_O28_Dakar	O28 serovar Dakar	hp-3306-B5R7B6	Gallinarum, Weltevreden
hp-3103-FL-g	f,g; f,g,s,t; f,g,t; g,m,p,q; g,m,s; g,m,s,t; g,m,t; g,t; g,z62	hp-3211-wzx_O28_Dakar	O28 serovar Dakar	hp-3307-B5R7C1	
hp-3104-FL-g	f,g; f,g,s,t; f,g,t; g,m,p,q; g,m,s; g,m,t; g,tg,z51	hp-3212-wzx_O28_Pomona	O28 serovar Pomona	hp-3308-ISR1	Infantis
hp-3105-FL-g	f,g; f,g,s,t; f,g,t; g,m,p,q; g,m,s; g,m,s,t; g,m,t; g,t; g,z51; g,z62;	hp-3213-wzx_O28_Pomona	O28 serovar Pomona	hp-3310-ISR1	Infantis
hp-3106-FL-g	f,g; f,g,s,t; f,g,t; g,m,p,q; g,m,s; g,m,s,t; g,m,t; g,t; g,z51; g,z62;	hp-3214-wzx_O3,10	O3,10	hp-3311-Q57QY4	Choleraesuis
hp-3107-FL-g	m,t	hp-3215-wzx_O3,10	O3,10	hp-3312-Q57QY4	Choleraesuis
hp-3108-FL-g	m,t	hp-3216-wzx_O30	O30	hp-3314-invA	species marker
hp-3109-FL-g	m,t	hp-3217-wzx_O30	O30	hp-3315-invA	species marker
hp-3113-FL-l+z39+z52	z39	hp-3218-wzx_O35	O35	hp-3316-invA	species marker

Flagellar probes were designed using distinctive antigenic sequences within phase 1 (*fliC*) and phase 2 (*fljB*) flagellar genes (Table 2). The following flagellar antigens can be identified on the array: a; b; c; d; e,h; e,n,x; e,n,x,z15; f,g; f,g,m,t; f,g,s; [f],g,[t]; f,g,t; g,[s],t; g,m; g,m,[p],s; g,m,[t]; g,m,q; g,m,s; g,m,s,t; g,m,t; g,p; g,p,s; g,p,u; g,q; g,s,t; g,t; g,z51; g,z62; i; j; k; (k); l,v; l,w; l,z13; l,z13,z28; l,z28; m,p,t,[u]; m,t; r; r,[i]; y; z; z10; z29; z35; z36; z36,z38; z38; z39; z4,z23; z4,z23,z32; z4,z24; z4,z32; z41; z44; z47; z52; z58; z6; z65; z69; z81; z91; 1,11 (AY353292); 1,16 (AY353263); 1,2; 1,[2],7; 1,2,7; 1,5; 1,5,(7); 1,5,7; 1,6; 1,7; e,n,x; e,n,x,z15; e,n,z15; k; l,w; l,z13,z28z; z10; z35; z39; z41; z50 and z6. Additionally, probes specifying *invA* [24], *galF* (this study) and *manC* (this study) that were introduced to confirm the identity of *Salmonella* and to serve as controls. These controls were always positive if *Salmonella* isolates were tested (Table 1). Further, two probes for the Vi capsular antigen (Table 2) were included and were partly positive for *S.* ser. Paratyphi C and always positive for *S*. ser. Typhi. All tested *S*. ser. Dublin strains were negative for the Vi capsular antigen.

Probes and primers for AMR genotyping of *Salmonella* serovars were derived from a genotyping microarray for *E. coli* that was previously developed, validated and described ([25], http://alere-technologies.com/fileadmin/Media/Paper/Ecoli/Supplement_Geue__layout_E_coli.xlsx).

77 probes were selected out of this panel because these genes, or variants thereof with a maximum of 2 mismatches, have previously been found in *Salmonella* serovars. The selected probes were used to detect the following antimicrobial resistance genes (Table 3): *aac3Ia*, *aac3Ie*, *aac6Ib*, *aac6II*, *aadA1*, *aadA2*, *aadA23b*, *aadA3*, *aadA5*, *aadB*, *ant2Ia*, *aphA*, *armA*, *sph*, *strA*, *strB* (resistance to various aminoglycosides); *catA1*, *catB3*, *catB8*, *cmlA*, *floR* (chloramphenicol); *tetA*, *tetB*, *tetC*, *tetD*, *tetG* (tetracyclines); *sul1*, *sul2*, *sul3*, *dfrA1*, *dfrA5*, *dfrA7*, *dfrA12*, *dfrA13*, *dfrA14*, *dfrA15*, *dfrA17*, *dfrA19* (sulfonamide/trimethoprim); *ble* (glycopeptides: bleomycin); *qnrA*, *qnrB*, *qnrD*, *qnrS* (quinolones); *acc1*, *carB2*, *cmy2*, *ctxM1*, *ctxM2*, *ctxM26*, *ctxM9*, *dha1*, *oxa1*, *oxa2, oxa10*, *oxa53*, *per2*, *shv*, *tem1* (beta-lactam compounds); *kpc4* (carbapenems) and *ereA*, *mphA* (macrolides). Additionally, two probes were designed to determine the presence of genes *intI1* and *intI2* possibly mediating an integrase function [26], [27].

**Table 3:** Summary of probes detecting antibiotic resistance genes and virulence factors.

Probe	Potential Function	Probe	Potential function
hp_armA_611	aminoglycoside resistance	hp_ble_611	bleomycin resistance
prob_aac3Ia_1	aminoglycoside resistance	prob_catA1_11	chloramphenicol resistance
hp_aac3_611	aminoglycoside resistance	prob_catB3_11	chloramphenicol resistance
prob_aac6Ib_1	aminoglycoside resistance	prob_catB8_12	chloramphenicol resistance
prob_aadA1_1	aminoglycoside resistance	prob_cmlA1_11	chloramphenicol resistance
prob_aadA2_1	aminoglycoside resistance	prob_floR_11	florfenicol and chloramphenicol resistance
prob_aadA4_1	aminoglycoside resistance	hp_mphA_611	erythomycin and roxythromycin resistance
prob_ant2Ia_1	aminoglycoside resistance	hp_ereA_611	erythromycin resistance
hp_aac6_612	aminoglycoside resistance	prob_qnrB_12	fluoroquinolone resistance
hp_aac6_615	aminoglycoside resistance	hp_kpc4_611	imipenem resistance
hp_aac6_618	aminoglycoside resistance	hp_qnrD_611	quinolone resistance
hp_aadB_611	aminoglycoside resistance	prob_qnr_12	quinolone resistance
hp_aadB-2_611	aminoglycoside resistance	prob_qnrS_11	quinolone resistance
hp_sph_611	aminoglycoside resistance	prob_sul1_11	sulfonamide resistance
prob_strA_611	aminoglycoside resistance	prob_sul2_11	sulfonamide resistance
prob_strB_611	aminoglycoside resistance	prob_sul3_11	sulfonamide resistance
hp_aac3_614	aminoglycoside resistance	prob_tetA_11	tetracycline resistance
hp_aphA_611	aminoglycoside resistance	prob_tetB_11	tetracycline resistance
hp_blaCMY_611	beta-lactam resistance	prob_tetC_11	tetracycline resistance
hp_per2_611	beta-lactam resistance	prob_tetD_1	tetracycline resistance
prob_acc1_11	beta-lactam resistance	prob_tetG_11	tetracycline resistance
prob_acc2_11	beta-lactam resistance	prob_dfr12_11	trimethoprim resistance
prob_cmy_11	beta-lactam resistance	prob_dfr13_11	trimethoprim resistance
prob_ctxM1_11	beta-lactam resistance	prob_dfrA1_21	trimethoprim resistance
prob_ctxM2_11	beta-lactam resistance	prob_dfrA1_22	trimethoprim resistance
prob_ctxM26_11	beta-lactam resistance	prob_dfrA14_21	trimethoprim resistance
prob_ctxM9_11	beta-lactam resistance	prob_dfrA15_1	trimethoprim resistance
prob_dha1_1	beta-lactam resistance	prob_dfrA17_11	trimethoprim resistance
prob_oxa1_21	beta-lactam resistance	prob_dfrA19_1	trimethoprim resistance
prob_oxa2_11	beta-lactam resistance	prob_dfrA7_11	trimethoprim resistance
prob_oxa7_11	beta-lactam resistance	prob_dfrA7_12	trimethoprim resistance
prob_per2_1	beta-lactam resistance	prob_dfrV_21	trimethoprim resistance
prob_pse1_1pm	beta-lactam resistance	prob_intI1_1	integrases
prob_shv1_11	beta-lactam resistance	prob_intI2_11	integrases
prob_tem1_1	beta-lactam resistance		

All 332 probes (255 serotyping and 77 AMR probes, synthesized by Metabion, Martinsried, Germany) were spotted at Alere Technologies, Germany in duplicates to the ArrayStrips as previously described [28]. Biotinylated oligonucleotides with a random sequence were spotted as a staining control and spotting buffer was spotted as a negative control.

### Multiplex linear DNA amplification and labeling for hybridization to prepared ArrayStrips

**Figure 1:**
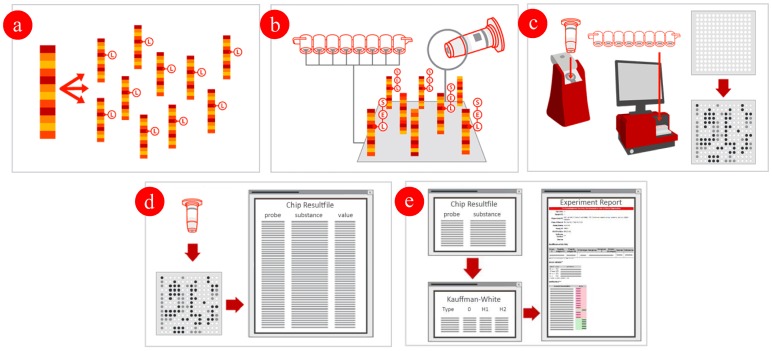
Multiplex linear DNA amplification, labeling and hybridization of the ArrayStrips. (a) Linear Multiplex Amplification starting from clonal RNA free genomic DNA, extracted DNA is internally labeled with biotin (Label [L]) and amplified in a linear multiplex PCR reaction; (b) Hybridization: the biotin labeled, single-stranded DNA product hybridizes specifically under stringent conditions to the corresponding probes. The resulting duplex is detected using a horse-radish peroxidase (Enzyme [E]) – streptavidin conjugate, which converts the substrate (Seramun green [S]) into a colored local precipitate. (c) Detection: the ArrayMate™ Reader (or ArrayTube™ Reader ATR 03) enables the visualization and subsequent automated analysis of the array image. The presence of a dark precipitated spot indicates successful hybridization; (d) Analysis: the assay specific software analysis script, supplied with the ArrayMate™ Reader (or ArrayTube™ Reader ATR 03), measures the signal intensity of each probe and determines with an assay specific algorithm which genes/alleles are present in the sample. (e) Genotype analysis: the PatternMatching software supplied with the ArrayMate™ Reader (or ArrayTube™ Reader ATR 03) is comparing the resulting pattern with a local database including 132 reference serovars previously sero- and genotyped, finally a report is given to which serovar the sample strain belongs with regard to the Kauffman-White Scheme.

For multiplex linear DNA amplification, a set of 292 primers (220 serotyping primer and 72 AMR primer, synthesized by Metabion, Martinsried, Germany) was used. These primers are located on the complementary strand, downstream of the sequence of the covalently immobilized oligonucleotide detection probes (the number of probes and primers do not need to be identical, a primer can target a consensus region, while probes might bind to more variable parts close by, which allows discerning different alleles of one gene). The labeling of the genomic DNA was accomplished during the linear amplification step by using dUTP linked biotin as a marker, thereby allowing site-specific internal labeling of the corresponding target region (Fig. 1a). Using the HybPlus Kit (Alere Technologies, Germany), at least 0.5 µg genomic DNA were labeled according to the manufacturer's instructions. The linear amplification steps included 5 min of initial denaturation at 96°C, followed by 50 cycles with 20 s of annealing at 50°C, 40 s of elongation at 72°C, and 60 s of denaturation at 96°C. This reaction results in a multitude of specifically amplified, single-stranded, biotin-labeled DNA molecules for subsequent hybridization to the corresponding DNA microarray.

### Hybridization of the ArrayStrips

For the hybridization procedures, the HybPlus Kit (Alere Technologies, Germany) was used according to the manufacturer's instructions with an adapted protocol. This included hybridization buffer C1, washing buffer C2, peroxidase-streptavidin conjugate C3, conjugation buffer C4, washing buffer C5 and peroxidase substrate D1.

First, ArrayStrips were placed in a thermomixer (Quantifoil Instruments, Jena, Germany) and subsequently washed with 200 µl of de-ionized water for 5 min at 55°C/550 rpm and with 100 µl hybridization buffer C1 for 5 min at 55°C/550 rpm. All liquids were always completely removed with a soft plastic pipette to avoid scratching of the chip surface. In a separate tube, 10 µl of the labeled, single-stranded DNA were dissolved in 90 µl hybridization buffer C1. The hybridization was carried out at 55°C, shaking at 550 rpm for 1 h. After hybridization, the ArrayStrips were washed two times for 5 min with 200 µl washing buffer C2 at 45°C, shaking at 550 rpm. Peroxidase-streptavidin conjugate C3 was diluted 1∶100 in buffer C4. A total of 100 µl of this mixture were added to each slot of the ArrayStrip, and subsequently incubated for 10 min at 30°C and 550 rpm. Afterwards, washing was carried out two times at 550 rpm with 200 µl C5 washing buffer at 30°C, with each step performed for 5 min. The visualization was achieved by adding 100 µl of peroxidase substrate D1 to the ArrayStrips, and signals were detected with the ArrayMate device (Alere Technologies, Jena, Germany) (Fig. 1b–c).

The described, final protocol was achieved by optimizing hybridization conditions (45°C–58°C) and washing temperatures (45°C–58°C) whereas the concentration of substances and incubation periods for each step were always constant. For this procedure, only strains were used for which published genome sequences (NCBI genome database) allowed to theoretically predict hybridization patterns (see Result part). These were strains *S*. ser. Agona (SL483), *S*. ser. Choleraesuis (SGSA50, SC-B67), *S*. ser. Dublin (SD3246, CT02021853), *S*. ser. Enteritidis (P125109), *S*. ser. Gallinarum (287/91, SG9), *S*. ser. Heidelberg (SL476), *S*. ser. Infantis (SIN), *S*. ser. Newport (SL254), *S*. ser. Paratyphi A (AKU_12601, ATCC9150), *S*. ser. Paratyphi B (SPB7), *S*. ser. Paratyphi C (RKS4594), *S*. ser. Schwarzengrund (CVM19633), *S*. ser. Typhi (CT18, Ty2), *S*. ser. Typhimurium (14028S, 27120, D23580, SL1344, T000240, UK-1, LT2) and *S*. ser. Weltevreden (2007-60-3289-1). These predictions were subsequently compared with the results of real hybridization experiments. The absence and presence of signals at different hybridization temperatures were monitored and the final protocol as described above based on the experiments in which the best accordance between predictions and real hybridizations was observed.

### Processing data using PatternMatch algorithm

Hybridization signals were processed using the IconoClust software, version 3.2r1 (Fig. 1d). All spots were normalized automatically by the software according to the quotation 
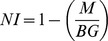
 where NI is the normalized intensity, M the average intensity of the automatically recognized spot, and BG the intensity of the local background. The output range of the signals were between 0 and 1 with 0 being negative and 1 being the maximal possible signal value. A probe-matching matrix was used to construct the theoretical hybridization pattern of the fully sequenced strains listed in NCBI database (Table S1). The definition of the theoretical signal intensity was 0.9 for perfect match, 0.6 for 1 mismatch, 0.3 for 2 mismatches, 0.1 and below for 3 mismatches and no signal for more mismatches. For each of these sequenced strains, at least one reference strain was used to assign the expected pattern with the pattern of the real hybridization experiments. For this operation, the PatternMatch algorithm was used [29]. The final numerical output was given as the matching score (MS), which represents the overall sum of all differences between corresponding signal intensities of theoretical and real hybridization experiments. Thus, the MS value is a measure of overall similarity/dissimilarity between two hybridization patterns. An ideal match of two patterns based on the same set of oligonucleotide probes will yield MS = 0, whereas values above MS = 6.5 require critical scrutiny because they may indicate a poor match. The Delta MS value, defined as the arithmetic difference between best and second best match, served as measure for the accuracy of species identification. A Delta MS higher than 1.5 was considered to be sufficient for an unambiguous distinction between two patterns.

Calculation of similarities was carried out by comparing signals for all 255 probes between theoretical predictions and real experiments. Signals with intensities higher than 0.3, were considered positive and set as “1”. Signals lower than 0.3, were regarded negative and set as “0”. The number of probe differences was summarized and the percentage was calculated. In order to assess the reproducibility, eight experiments were performed under identical conditions. All experiments were compared to each other using the PatternMatch algorithm and the mean, maximum and minimum MS were calculated.

### Antimicrobial resistance

All isolates in which AMR genes were detected, a total of 34 *Salmonella* isolates belonging to 18 serovars, were tested for their phenotypic antimicrobial resistance. This was carried out using the VITEK 2 system with the AST-N111 test panel (bioMérieux Deutschland GmbH, Nürtingen, Germany). Additionally, chloramphenicol (30 µg), kanamycin (30 µg) and streptomycin (10 µg) were tested by disk diffusion assay. This assay was performed using CLSI.

### Field study

A panel of 105 *Salmonella* isolates was tested in a blinded field study. All isolates were serotyped using the standard procedures [12] at the National Reference Laboratory for Salmonellosis in Cattle at the Friedrich-Loeffler-Institute (Jena, Germany). The serotyped isolates were subsequently genotyped using the automated PatternMatch software installed on the ArrayMate device (IconoClust version 3.2r1). Finally, serotyping and genotyping results were compared. Within this blinded panel, mistakes were defined as “major”, if a serovar was completely falsely identified (*e.g.*, a Dublin isolate as Naestved). A “minor” mistake was if the serovar was identified correctly, but if a variant of this serovar was not recognized (*e.g.*, *S.* ser. Typhimurium var. Copenhagen as *S.* ser. Typhimurium). Per definition, only “major” mistakes were regarded as incorrect hits.

## Results

### Verification of the assay and database building for PatternMatch

**Figure 2:**
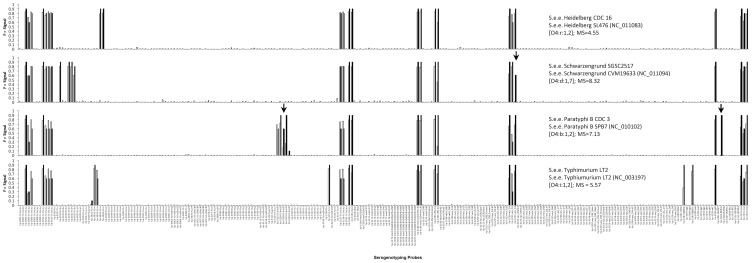
Comparison for four different Salmonella serovars between theoretical predictions (white) and real hybridizations (black). Arrows indicate the probes where differences were observed. For strain S. ser. Typhimurium LT2, the antigenic formula, the complete genome sequence (NC_003197.1), and a prediction for its hybridization pattern based on this sequence were available. The threshold matching score (MS) of 6.5 between two real hybridization experiments do not apply for this comparison, as theoretical predictions do not represent real results.

A set of 168 *Salmonella* strains representing 132 different serovars were used to evaluate the probes printed on the array, the primers in the labeling mixture, and to build a database for identification of the globally most prominent *Salmonella* serovars. Comparison of predicted and real hybridization results was performed for strains with fully sequenced genomes (see Materials and Methods and Table 4). The similarity between the predicted and real hybridization results of the serogenotyping array was more than 99 percent (Table 4). Because both, the full sequence information of the genome and the antigenic formula of *S.* ser. Typhimurium strain LT2, were available, an exact comparison of predicted and actual experimental hybridization pattern was possible (Fig. 2). It showed a 100% identity when regarding just positive and negative signals. A more detailed analysis, also considering signal intensities, showed a high degree of similarity between theoretical predictions and actual experiments with exceptions at probe hp-3221-wzx_O4 (signal intensity increased about 42% as predicted by the theoretical experiment) and hp-3282-Q8ZK10 (signal intensity decreased about 43% as predicted by the theoretical experiment). The highest discrepancy was found for *S*. ser. Paratyphi A and *S*. ser. Paratyphi B. Analysis of the results of *S*. ser. Paratyphi B showed that two probes were negative in actual hybridizations compared to the theoretical predictions (Fig. 2). However, the missing probes were redundant for one target gene (*e.g.*; *S*. ser. Paratyphi B *fliC*-H1:b), so that this issue did not influence the identification. Because of the high correlation between theoretical predictions and actual experiments, as well as the high similarity of T_m_ of all 255 serotyping probes, it is assumed that the detection efficiency with other *Salmonella* serovars will also be comparably precise under the same conditions. Furthermore, the results of these theoretical experiments were used to find an optimal protocol (data not shown) for the hybridization of the *Salmonella* array so that an optimal, stringent hybridization and washing temperature could be defined (see Methods part).

**Table 4:** Comparison of theoretical predictions and real hybridization patterns for the *Salmonella* array and previously typed strains (CDC, DSMZ).

Virtual Hybridization						Real Hybridization			Comparison	
Serovar	Strain	Accession No.	Serogroup	Antigenic Formula	Correct Antigenic Formula Designation	Serovar	Reference Strain	Correct Serovar Designation	Number of Probe Differences between Virtual and Real Hybridization^a^	Similarity in %
Agona	SL483	NC_011149.1	B (O:4)	1,4,[5],12:f,g,s:[1,2]	YES	Agona	CDC1636	YES	0/255	100.0
Choleraesuis	SGSA50	CM001062.1	C1 (O:7)	6,7:c:1,5	YES	Choleraesuis	DSM 14846	YES	0/255	100.0
Choleraesuis	SC-B67	NC_006905	C1 (O:7)	6,7:c:1,5	YES	Choleraesuis	DSM14846	YES	0/255	100.0
Dublin	SD3246	CM001151.1	D1 (O:9)	1,9,12[Vi]:g,p:-	YES	Dublin	CDC10-0635	YES	1/255	99.6
Dublin	CT02021853	NC_011205.1	D1 (O:9)	1,9,12[Vi]:g,p:-	YES	Dublin	CDC10-0636	YES	1/255	99.6
Enteritidis	P125109	NC_011294.1	D1 (O:9)	1,9,12:g,m:-	YES	Enteritidis	DSM17420	YES	0/255	100.0
Gallinarum	287/91	NC_011274.1	D1 (O:9)	1,9,12:-:-	YES	Gallinarum	CDC74	YES	1/255	99.6
Gallinarum	SG9	CM001153.1	D1 (O:9)	1,9,12:-:-	YES	Gallinarum	CDC74	YES	1/255	99.6
Heidelberg	SL476	NC_011083.1	B (O:4)	1,4,[5],12:r:1,2	YES	Heidelberg	CDC16	YES	0/255	100.0
Infantis	SIN	sanger.ac.uk^b^	C1 (O:7)	6,7,14:r:1,5	YES	Infantis	CDC1428	YES	0/255	100.0
Newport	SL254	NC_011080.1	C2–C3 (O:8)	6,8,20:e,h:1,2	YES	Newport	CDC 2434	YES	0/255	100.0
Paratyphi A	AKU_12601	NC_011147.1	A (O:2)	1,2,12:a:[1,5]	YES	Paratyphi A	CDC1	YES	2/255	99.2
Paratyphi A	ATC C9150	NC_006511.1	A (O:2)	1,2,12:a:[1,5]	YES	Paratyphi A	CDC1	YES	2/255	99.2
Paratyphi B	SPB7	NC_010102.1	B (O:4)	1,4,[5],12:b:1,2	YES	Paratyphi B	CDC3	YES	2/255	99.2
Paratyphi C	RKS4594	NC_012125.1	C1 (O:7)	6,7,[Vi]:c:1,5	YES	Paratyphi C	CDC33	YES	0/255	100.0
Schwarzengrund	CVM19633	NC_011094.1	B (O:4)	1,4,12,27:d:1,7	YES	Schwarzengrund	CDC1629	YES	1/255	99.6
Typhi	CT18	NC_003198.1	D1 (O:9)	9,12[Vi]:d:-	YES	Typhi	No. 1^c^	YES	0/255	100.0
Typhi	Ty2	NC_004631.1	D1 (O:9)	9,12[Vi]:d:-	YES	Typhi	No. 1^c^	YES	0/255	100.0
Typhimurium	14028S	NC_016856.1	B (O:4)	1,4,[5],12:i:1,2	YES	Typhimurium	CDC14	YES	0/255	100.0
Typhimurium	27120	NC_016857.1	B (O:4)	1,4,[5],12:i:1,2	YES	Typhimurium	CDC14	YES	0/255	100.0
Typhimurium	D23580	FN424405.1	B (O:4)	1,4,[5],12:i:1,2	YES	Typhimurium	CDC14	YES	0/255	100.0
Typhimurium	LT2	NC_003197.1	B (O:4)	1,4,[5],12:i:1,2	YES	Typhimurium	LT2	YES	0/255	100.0
Typhimurium	SL1344	NC_016810.1	B (O:4)	1,4,[5],12:i:1,2	YES	Typhimurium	CDC14	YES	0/255	100.0
Typhimurium	T000240	NC_016860.1	B (O:4)	1,4,[5],12:i:1,2	YES	Typhimurium	CDC14	YES	0/255	100.0
Typhimurium	UK-1	NC_016863.1	B (O:4)	1,4,[5],12:i:1,2	YES	Typhimurium	CDC14	YES	0/255	100.0
Weltevreden	2007-60-3289-1	FR775255.1	E1 (O:3,10)	3,{10}{15}:r:z6	YES	Weltevreden	CDC147	YES	0/255	100.0

a maximal difference of serogenotyping probes at a signal threshold of 0.3.

b ftp://ftp.sanger.ac.uk/pub/pathogens/Salmonella/SG.dbs.

c only genomic DNA of *Salmonella* Typhi, courtesy of Rene S. Hendriksen, DTU Food, Denmark.

Calculation of similarities was carried out by comparing predictions to measured signals for all 255 probes. Signals with intensities higher than 0.3, were considered positive and set as “1”. Signals lower than 0.3 were regarded negative and set as “0”. The number of probes which differ was summarized and the percentage was calculated.

Using this optimized protocol (as described in Materials and Methods), strains of all 132 *Salmonella* serovars were analyzed Each serovar was tested at least three times using the *Salmonella* array to ensure consistent results and the identification of the unique and reproducible serovar-specific probe patterns. These unique patterns were used to build a PatternMatch database consisting of data from real experiments instead of theoretical experiments from defined strains. A manual serotyping using the probe-function table (Table 2) was restricted by the resolution of probes identify the H2-phase. This phase was mainly a combination of different probes, *e.g.* H2:1,5 of different serovars was always a combination of different “FL-1+e,n,x” probes (Table 2). Nevertheless it was possible to estimate the *Salmonella* serotype at least for the serogroup and in most cases for the phase H1. In the end the probe-function table served as a control for classical serotyped *Salmonella* before they were used in the PatterMatch database.

### Detection software

**Figure 3:**
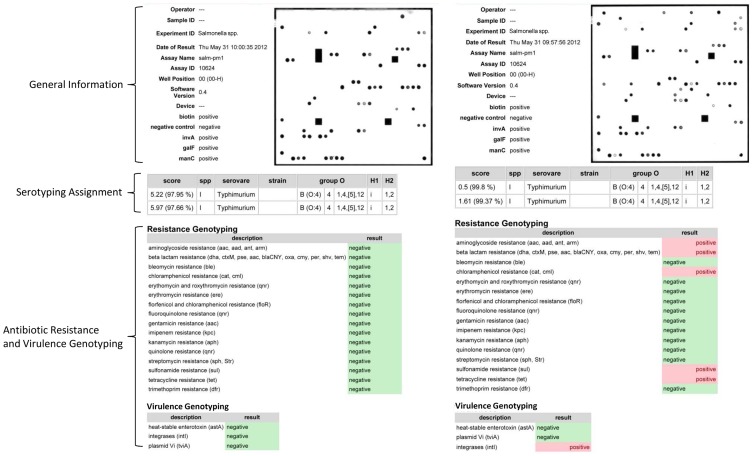
Result sheets of the PatternMatch software as generated by the ArrayMateTM Reader. General information: sample ID, negative/positive staining control and marker detecting genus Salmonella (invA, galf, manC). Serotyping assignment section: two best hits according to the processed sample were given as matching scores (MS) and as percentage. Antibiotic resistance and virulence genotyping section: detectable resistance and virulence genes. The pattern looks slightly different because of positive probes for resistance genes (aadA1, aadA2, cmlA1, dfrA12, sul3, tem1, tetB) that usually are located on mobile genetic elements.

Using the described PatternMatch module, a software package was developed to analyze *Salmonella* serovars directly at the ArrayMate device directly after scanning and calculating signals of the stained arrays (IconoClust Software version 3.2r1) (Fig. 1e). The detection software used the same database comprising 168 reference *Salmonella* strains (representing 132 *Salmonella* serovars) which were classically serotyped. Patterns of unknown *Salmonella* were compared to the whole database and the two best hits were given in a result sheet (Fig. 3). Prior to PatternMatching, all calculated signals were normalized within a range of 0 and 1. Briefly, the mean of valid signals was calculated and subsequently, the formula
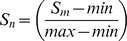
 (*S_n_* = normalized signal, *S_m_* = mean of signal, *min* = Minimum of all signals, *max* = maximum of all signals) was used to normalize the mean of valid values. Due to the normalization procedure, experiments with very low signal intensity could also be analyzed and subsequently compared with the database. This method guaranteed a correct assignment to the reference pattern within the provided database. Furthermore, different parameters were requested by the software: a) two biotin marker spots as positive staining controls, b) spotting buffer as a negative control and c) marker for detection of *Salmonella*. These results were included in the result sheet (Fig. 3). Additionally, the report contains the genotyping results of all AMR genes. The software tool was evaluated using all reference strains included in the database. All 168 reference strains were perfectly identified even if the experiment showed week signals (data not presented here). A multiple PatternMatch analysis of eight identical hybridization experiments with the same genomic DNA isolated from *S.* ser. Typhimurium DSM5569 showed a mean matching score (MS) of 2.12±0.65 with a maximum MS of 3.32 and a minimum MS of 1.15. The mean and maximum MS were significantly (t-test, p<0.05) lower than the MS value for poor matches (MS> = 6.5). These results showed the high reproducibility of this assay described in this study.

### Antibiotic resistance

In a panel of 34 *Salmonella* strains 26 different AMR genes were detected and subsequently compared with the AMR phenotype of these strains (Table 5, detailed view in Table S2). A high correlation was observed for all detected genes relating to the AMR phenotype.

**Table 5:** Comparison of antimicrobial resistance (AMR) genotype and AMR phenotype.

AMR Genes	Genbank No.	AMR Family	Antibiotics Tested to AMR Phenotype	Gene detected	Resistance dedected	Sensitivity detected	Correlation (%)
*aac6II*	AY123251.1	Aminoglycoside	Gentamicin, Tobramycin	1	1	0	100
*aadA1*	AB126599.1	Aminoglycoside	Streptomycin	14	14	0	100
*aadA2*	AB126602.1	Aminoglycoside	Streptomycin	5	5	0	100
*aphA1*	AB366440.1	Aminoglycoside	Kanamycin	5	5	0	100
*sph*	AB366441.1	Aminoglycoside	Streptomycin	1	1	0	100
*strA*	AB366440.1	Aminoglycoside	Streptomycin	10	10	0	100
*strB*	AB366440.1	Aminoglycoside	Streptomycin	14	14	0	100
*catA1*	AB366440.1	Chloramphenicol	Chloramphenicol	5	5	0	100
*cmlA*	AJ487033.2	Chloramphenicol	Chloramphenicol	4	4	0	100
*floR*	AF118107.1	Chloramphenicol	Chloramphenicol	2	2	0	100
*sul1*	AF261825.2	Sulfonamide/Trimethoprim	Co-trimoxazol (Sulfamethoxazol/Trimethoprim)	8	1	7	-a
*sul2*	AB366440.1	Sulfonamide/Trimethoprim	Co-trimoxazol (Sulfamethoxazol/Trimethoprim)	3	2	1	-a
*sul1*, *sul2*	AF261825.2, AB366440.1	Sulfonamide/Trimethoprim	Co-trimoxazol (Sulfamethoxazol/Trimethoprim)	1	0	1	-a
*sul1*, *dfrA1*	AF261825.2, AF203818.1	Sulfonamide/Trimethoprim	Co-trimoxazol (Sulfamethoxazol/Trimethoprim)	1	1	0	100
*sul1*, *dfrA1*, *dfrA15*	AF261825.2, AF203818.1, AJ867237.1	Sulfonamide/Trimethoprim	Co-trimoxazol (Sulfamethoxazol/Trimethoprim)	1	1	0	100
*sul1*, *dfrA12*	AF261825.2, AB366440.1	Sulfonamide/Trimethoprim	Co-trimoxazol (Sulfamethoxazol/Trimethoprim)	1	1	0	100
*sul1*, *dfrA13*	AF261825.2, AM932669.1	Sulfonamide/Trimethoprim	Co-trimoxazol (Sulfamethoxazol/Trimethoprim)	1	1	0	100
*sul1*, *dfrV*	AF261825.2, DQ133160.1	Sulfonamide/Trimethoprim	Co-trimoxazol (Sulfamethoxazol/Trimethoprim)	1	1	0	100
*sul1*, *sul2*, *dfrA1*	AF261825.2, AB366440.1, AF203818.1	Sulfonamide/Trimethoprim	Co-trimoxazol (Sulfamethoxazol/Trimethoprim)	2	2	0	100
*sul1*, *sul2*, *dfrA12*	AF261825.2, AB366440.1, AB366440.1	Sulfonamide/Trimethoprim	Co-trimoxazol (Sulfamethoxazol/Trimethoprim)	1	1	0	100
*sul2*, *dfrA14*	AB366440.1, DQ388123.1	Sulfonamide/Trimethoprim	Co-trimoxazol (Sulfamethoxazol/Trimethoprim)	1	1	0	100
*sul3*, *dfrA1*	AY316203.1, AF203818.1	Sulfonamide/Trimethoprim	Co-trimoxazol (Sulfamethoxazol/Trimethoprim)	1	1	0	100
*sul3*, *dfrA12*	AY316203.1, AB366440.1	Sulfonamide/Trimethoprim	Co-trimoxazol (Sulfamethoxazol/Trimethoprim)	3	3	0	100
*ctxM1*	FJ654733.1	Beta-Lactam	Ampicillin, Piperacillin, Cefuroxime, Cefpodoxime, Ceftazidime	1	1	0	100
*oxa1*	AB218659.1	Beta-Lactam	Ampicillin, Piperacillin, Cefuroxime	2	2	0	100
*tem1*	AB366440.1	Beta-Lactam	Ampicillin, Piperacillin	15	15	0	100
*tetA*	AB366441.1	Tetracycline	Tetracycline	10	10	0	100
*tetB*	AB366440.1	Tetracycline	Tetracycline	13	13	0	100
*tetG*	AF261825.2	Tetracycline	Tetracycline	1	1	0	100
*mphA*	AY333434.1	Macrolide		1	-	-	-b
no gene detected		Aminoglycoside	Streptomycin	0	2	0	-c
no gene detected		Beta-Lactam	Ampicillin	0	1	0	-c

a only co-trimoxazol was tested, sulfamethoxazol was not available.

b potential resistance against erythromycin was not tested.

c AMR phenotype without AMR genotype was detected.

All AMR genes detected in 34 *Salmonella* strains listed and compared with the AMR phenotype. The phenotype was defined using a VITEK 2 system with an AST-N111 panel and a disk diffusion assay using Oxoid discs with chloramphinicol (30 mg), streptomycin (10 mg) and kanamycin (30 mg).

An extended-spectrum beta-lactamase (ESBL) gene, *ctxM1,* was detected once, in an isolate of *S.* ser. Anatum AMR07. This strain was resistant against ceftazidime and cefpodoxime, both members of third generation beta lactams.

AMR phenotypes for which no corresponding AMR genotype were detected included streptomycin resistance in two isolates (*S*. ser. Saintpaul and *S*. ser. 1,4,[5],12:i:-) and ampicillin resistance in one *S*. ser Bredeney isolate. The latter isolate yielded a positive signal in a nitrocefin assay (BBL DrySlide Nitrocefin, Becton Dickinson).

No assessment was possible for resistance genes *sul1* and *sul2* that should cause isolated resistance to sulfonamides because testing was performed only for co-trimoxacole only.). A gene *mphA* mediating erythromycin resistance was found in *S*. ser. Anatum strain AMR05 (Table S2), but erythromycin susceptibility was not tested using a panel for gram-negatives on the VITEK 2 system.

### Field study

After assay verification, the *Salmonella* serogenotyping assay was used to identify a field panel of 105 *Salmonella* isolates (Table 6) sampled and serotyped by the National Reference Laboratory for Salmonellosis in Cattle at the Friedrich-Loeffler-Institute (FLI, Jena, Germany). All tested isolates were identified as *Salmonella* and, out of 105 isolates, 93 were typed correctly (88.6%, Table 6). The limitation of the actual assay was that certain strains yielded identical patterns on the current array thus prohibiting further differentiation (Table 6). Such limitations occurred for *S*. ser. Enteritidis, which actually cannot be discriminated from *S*. ser. Nitra and *S*. ser. Blegdam. Furthermore, the pattern of *S*. ser. Dublin was identical to *S*. serovars Naestved, Moscow and Kiel. A discrimination of *S*. ser. Dublin and *S*. ser. Kiel was impossible as probes representing the genes *SeD_A1100*, *SeD_A1101 and SeD_A1102* were positive for both serovars. Similar limitations were also observed for *S*. ser. Panama (identical pattern as *S*. ser. Koessen), *S*. ser. Indiana (identical pattern as *S*. ser. Kiambu) and *S*. ser. Senftenberg (identical pattern as *S*. ser. Westhampton). A monophasic *S*. ser. Typhimurium isolate (1,4,[5],12:i:-) was identified correctly. *Salmonella* ser. Typhimurium var. Copenhagen (1,4,12:i:1,2) was assigned to *S*. ser. Typhimurium (1,4,[5],12:i:1,2) and a rough form of *S*. ser. Infantis was assigned to non-rough *S*. ser. Infantis. These limitations were evaluated as minor mistakes and subsequently regarded as correct hits.

**Table 6:** Field study with a blind panel of 105 isolates using the *Salmonella* serogenotyping assay.

Species	Serovar	Strain	Results of classical Serotyping		Results of microarray based Serotyping		
			Serogroup	Antigenic Formula	Unique Pattern	Serovar	Alternative Serovar^a^
*S.e. enterica*	1,4,5,12:i:-	NRL688	B (O:4)	1,4,[5],12:i:-	yes	1,4,[5],12:i:-	
*S.e. enterica*	1,4,5,12:i:-	NRL749	B (O:4)	1,4,[5],12:i:-	yes	1,4,[5],12:i:-	
*S.e. enterica*	1,4,5,12:i:-	NRL813	B (O:4)	1,4,[5],12:i:-	yes	1,4,[5],12:i:-	
*S.e. enterica*	1,4,5,12:i:-	NRL982	B (O:4)	1,4,[5],12:i:-	yes	1,4,[5],12:i:-	
*S.e. enterica*	1,4,5,12:i:-	NRL1004	B (O:4)	1,4,[5],12:i:-	yes	1,4,[5],12:i:-	
*S.e. enterica*	1,4,5,12:i:-	NRL1019	B (O:4)	1,4,[5],12:i:-	yes	1,4,[5],12:i:-	
*S.e. enterica*	Abony	NRL794	B (O:4)	1,4,[5],12,[27]:b:e,n,x	yes	Abony	
*S.e. enterica*	Agona	FLI415	B (O:4)	1,4,[5],12:f,g,s:[1,2]	yes	Agona	
*S.e. enterica*	Agona	FLI417	B (O:4)	1,4,[5],12:f,g,s:[1,2]	yes	Agona	
*S.e. enterica*	Agona	FLI1157	B (O:4)	1,4,[5],12:f,g,s:[1,2]	yes	Agona	
*S.e. enterica*	Agona	FLI449	B (O:4)	1,4,[5],12:f,g,s:[1,2]	yes	Agona	
*S.e. enterica*	Agona	FLI709	B (O:4)	1,4,[5],12:f,g,s:[1,2]	yes	Agona	
*S.e. enterica*	Agona	FLI1027	B (O:4)	1,4,[5],12:f,g,s:[1,2]	yes	Agona	
*S.e. enterica*	Anatum	NRL939	E1 (O:3,10)	3,{10}{15}{15,34}:e,h:1,6	yes	Anatum	
*S.e. enterica*	Anatum	NRL946	E1 (O:3,10)	3,{10}{15}{15,34}:e,h:1,6	yes	Anatum	
*S.e. enterica*	Anatum	FLI452	E1 (O:3,10)	3,{10}{15}{15,34}:e,h:1,6	yes	Anatum	
*S.e. enterica*	Anatum	NRL1006	E1 (O:3,10)	3,{10}{15}{15,34}:e,h:1,6	yes	Anatum	
*S.e. enterica*	Bareilly	FLI608	C1 (O:7)	6,7,14:y:1,5	yes	Bareilly	
*S.e. enterica*	Bovismorbificans	FLI646	C2–C3 (O:8)	6,8,20:r,[i]:1,5	yes	Bovismorbificans	
*S.e. enterica*	Bovismorbificans	FLI525	C2–C3 (O:8)	6,8,20:r,[i]:1,5	yes	Bovismorbificans	
*S.e. enterica*	Braenderup	FLI544	C1 (O:7)	6, 7,14: e,h: e,n,z15	yes	Braenderup	
*S.e. enterica*	Brandenburg	NRL796	B (O:4)	4,[5],12:l,v:e,n,z15	yes	Brandenburg	
*S.e. enterica*	Brandenburg	NRL869	B (O:4)	4,[5],12:l,v:e,n,z15	yes	Brandenburg	
*S.e. enterica*	Brandenburg	NRL892	B (O:4)	4,[5],12:l,v:e,n,z15	yes	Brandenburg	
*S.e. enterica*	Brandenburg	FLI419	B (O:4)	4,[5],12:l,v:e,n,z15	yes	Brandenburg	
*S.e. enterica*	Cerro	NRL721	K (O:18)	6,14,18:z4,z23:[1,5]	yes	Cerro	
*S.e. enterica*	Choleraesuis	FLI826	C1 (O:7)	6,8:c:1,6	yes	Choleraesuis	
*S.e. enterica*	Choleraesuis	FLI987	C1 (O:7)	6,7:c:1,5	yes	Choleraesuis	
*S.e. enterica*	Derby	FLI605	B (O:4)	1,4,[5],12:f,g:[1,2]	yes	Derby	
*S.e. enterica*	Derby	FLI624	B (O:4)	1,4,[5],12:f,g:[1,2]	yes	Derby	
*S.e. enterica*	Derby	NRL723	B (O:4)	1,4,[5],12:f,g:[1,2]	yes	Derby	
*S.e. enterica*	Derby	NRL776	B (O:4)	1,4,[5],12:f,g:[1,2]	yes	Derby	
*S.e. enterica*	Derby	NRL960	B (O:4)	1,4,[5],12:f,g:[1,2]	yes	Derby	
*S.e. enterica*	Derby	FLI529	B (O:4)	1,4,[5],12:f,g:[1,2]	no	Derby	
*S.e. enterica*	Derby	FLI624	B (O:4)	1,4,[5],12:f,g:[1,2]	yes	Derby	
*S.e. enterica*	Derby	FLI666	B (O:4)	1,4,[5],12:f,g:[1,2]	yes	Derby	
*S.e. enterica*	Derby	FLI1111	B (O:4)	1,4,[5],12:f,g:[1,2]	no	Derby	
*S.e. enterica*	Dublin	NRL683	D1 (O:9)	1,9,12[Vi]:g,p:-	no	Moscow	Naestved
*S.e. enterica*	Dublin	NRL684	D1 (O:9)	1,9,12[Vi]:g,p:-	no	Naestved	Dublin
*S.e. enterica*	Dublin	NRL703	D1 (O:9)	1,9,12[Vi]:g,p:-	no	Naestved	Dublin
*S.e. enterica*	Dublin	NRL704	D1 (O:9)	1,9,12[Vi]:g,p:-	no	Naestved	Dublin
*S.e. enterica*	Dublin (Bovisaloral)	NRL915	D1 (O:9)	1,9,12[Vi]:g,p:-	no	Naestved	Dublin
*S.e. enterica*	Dublin (rough)	NRL904	D1 (O:9)	1,9,12[Vi]:g,p:-	no	Naestved	Dublin
*S.e. enterica*	Enteritidis	FLI95	D1 (O:9)	1,9,12:g,m:-	no	Blegdam	Enteritidis
*S.e. enterica*	Enteritidis	NRL685	D1 (O:9)	1,9,12:g,m:-	no	Nitra	Enteritidis
*S.e. enterica*	Enteritidis	NRL875	D1 (O:9)	1,9,12:g,m:-	no	Belgdam	Enteritidis
*S.e. enterica*	Gallinarum	FLI151	D1 (O:9)	1,9,12:-:-	yes	Gallimarum	
*S.e. enterica*	Gallinarum	FL155	D1 (O:9)	1,9,12:-:-	yes	Gallimarum	
*S.e. enterica*	Gallinarum	FLI969	D1 (O:9)	1,9,12:-:-	yes	Gallimarum	
*S.e. enterica*	Goldcoast	NRL852	C2–C3 (O:8)	6,8:r:l,w	yes	Goldcoast	
*S.e. enterica*	Goldcoast	FLI990	C2–C3 (O:8)	6,8:r:l,w	yes	Goldcoast	
*S.e. enterica*	Hadar	FLI636	C2–C3 (O:8)	6,8:z10:e,n,x	yes	Hadar	
*S.e. enterica*	Hadar	FLI638	C2–C3 (O:8)	6,8:z10:e,n,x	yes	Hadar	
*S.e. enterica*	Hadar	FLI672	C2–C3 (O:8)	6,8:z10:e,n,x	yes	Hadar	
*S.e. enterica*	Havana	FLI607	G (O:13)	1,13,23:f,g,[s]:-	yes	Havanna	
*S.e. enterica*	Havana	FLI575	G (O:13)	1,13,23:f,g,[s]:-	yes	Havana	
*S.e. enterica*	Indiana	NRL872	B (O:4)	1,4,12:z:1,7	no	Kiambu	Indiana
*S.e. enterica*	Infantis	NRL718	C1 (O:7)	6,7,14:r:1,5	yes	Infantis	
*S.e. enterica*	Infantis	NRL822	C1 (O:7)	6,7,14:r:1,5	yes	Infantis	
*S.e. enterica*	Infantis	FLI630	C1 (O:7)	6,7,14:r:1,5	yes	Infantis	
*S.e. enterica*	Infantis	FLI761	C1 (O:7)	6,7,14:r:1,5	yes	Infantis	
*S.e. enterica*	Infantis (R-form)	FLI546	C1 (O:7)	6,7,14:r:1,5	yes	Infantis	
*S.e. enterica*	Kaseneyi	NRL878	P (O:38)	38:e,h:1,5	yes	Kaseneyi	
*S.e. enterica*	Kedougou	FLI515	G (O:13)	1,13,23:i:l,w	yes	Kedougou	
*S.e. enterica*	Kedougou	NRL1022	G (O:13)	1,13,23:i:l,w	yes	Kedougou	
*S.e. enterica*	Litchfield	FLI1218	C2–C3 (O:8)	6,8:l,v:1,2	yes	Litchfield	
*S.e. enterica*	Livingstone	FLI720	C1 (O:7)	6,7,14:d:l,w	yes	Livingstone	
*S.e. enterica*	London	NRL700	E1 (O:3,10)	3,{10}{15}:l,v:1,6	yes	London	
*S.e. enterica*	London	NRL849	E1 (O:3,10)	3,{10}{15}:l,v:1,6	yes	London	
*S.e. enterica*	Manhattan	FLI662	C2–C3 (O:8)	6,8:d:1,5	yes	Manhattan	
*S.e. enterica*	Mbandaka	FLI534	C1 (O:7)	6,7,14:z10:e,n,z15	yes	Mbandaka	
*S.e. enterica*	Minnesota	NRL814	L (O:21)	21:b:e,n,x	yes	Minnesota	
*S.e. enterica*	Minnesota	NRL839	L (O:21)	21:b:e,n,x	yes	Minnesota	
*S.e. enterica*	Montevideo	NRL930	C1 (O:7)	6,7,14:g,m,[p],s:[1,2,7]	yes	Montevideo	
*S.e. enterica*	Montevideo	FLI652	C1 (O:7)	6,7,14:g,m,[p],s:[1,2,7]	yes	Montevideo	
*S.e. enterica*	Muenchen	NRL801	C2–C3 (O:8)	6,8:d:1,2	yes	Muenchen	
*S.e. enterica*	Muenster	FLI325	E1 (O:3,10)	3,{10}{15}{15,34}:e,h:1,5	yes	Muenster	
*S.e. enterica*	Ohio	NRL882	C1 (O:7)	6,7,14:b:l,w	yes	Ohio	
*S.e. enterica*	Oranienburg	FLI429	C1 (O:7)	6,7,14:m,t:[z57]	yes	Oranienburg	
*S.e. enterica*	Panama	FLI604	D1 (O:9)	1,9,12:l,v:1,5	no	Koessen	Panama
*S.e. enterica*	Panama	FLI411	D1 (O:9)	1,9,12:l,v:1,5	no	Panama	Koessen
*S.e. enterica*	Panama	FLI413	D1 (O:9)	1,9,12:l,v:1,5	no	Panama	Koessen
*S.e. enterica*	Paratyphi B	FLI588	B (O:4)	1,4,[5],12:b:1,2	yes	Paratyphi B	
*S.e. enterica*	Paratyphi B	FLI590	B (O:4)	1,4,[5],12:b:1,2	yes	Paratyphi B	
*S.e. enterica*	Pomona	FLI700	M (O:28)	28:y:1,7	yes	Pomona	
*S.e. enterica*	Saintpaul	FLI344	B (O:4)	1,4,[5],12:e,h:1,2	yes	Saintpaul	
*S.e. enterica*	Saintpaul	FLI423	B (O:4)	1,4,[5],12:e,h:1,2	yes	Saintpaul	
*S.e. enterica*	Sandiego	NRL987	C1 (O:7)	1,4,[5],12:e,h:e,n,z15	yes	Sandiego	
*S.e. enterica*	Senftenberg	NRL682	E4 (O:1,3,19)	1,3,19:g,[s],t:-	no	Westhampton	Senftenberg
*S.e. enterica*	Tennessee	FLI347	C1 (O:7)	6,7,14:z29:[1,2,7]	yes	Tennessee	
*S.e. enterica*	Tennessee	FLI606	C1 (O:7)	6,7,14:z29:[1,2,7]	yes	Tennessee	
*S.e. enterica*	Thompson	FLI658	C1 (O:7)	6,7,14:k:1,5	yes	Thompson	
*S.e. enterica*	Typhimurium	FLI598	B (O:4)	1,4,[5],12:i:1,2	yes	Typhimurium	
*S.e. enterica*	Typhimurium	NRL990	B (O:4)	1,4,[5],12:i:1,2	yes	Typhimurium	
*S.e. enterica*	Typhimurium	NRL729	B (O:4)	1,4,[5],12:i:1,2	yes	Typhimurium	
*S.e. enterica*	Typhimurium	NRL737	B (O:4)	1,4,[5],12:i:1,2	yes	Typhimurium	
*S.e. enterica*	Typhimurium	FLI617	B (O:4)	1,4,[5],12:i:1,2	yes	Typhimurium	
*S.e. enterica*	Typhimurium	NRL990	B (O:4)	1,4,[5],12:i:1,2	yes	Typhimurium	
*S.e. enterica*	Typhimurium	NRL993	B (O:4)	1,4,[5],12:i:1,2	yes	Typhimurium	
*S.e. enterica*	Typhimurium	NRL1000	B (O:4)	1,4,[5],12:i:1,2	yes	Typhimurium	
*S.e. enterica*	Typhimurium var. Copenhagen	NRL797	B (O:4)	1,4,12:i:1,2	yes	Typhimurium	
*S.e. enterica*	Typhimurium var. Copenhagen	NRL912	B (O:4)	1,4,12:i:1,2	yes	Typhimurium	
*S.e. enterica*	Typhimurium var. Copenhagen	FLI1033	B (O:4)	1,4,12:i:1,2	yes	Typhimurium	
*S.e. enterica*	Virchow	FLI640	C1 (O:7)	6,7,14:r:1,2	yes	Virchow	
*S.e. enterica*	Virchow	FLI649	C1 (O:7)	6,7,14:r:1,2	yes	Virchow	

a tested isolate generated a serogenotyping pattern which is shared by multiple serovars.

Results were analyzed by the PatternMatch software and compared with the results of classical serotyping performed by the National Reference Laboratory for Salmonellosis in cattle at the Friedrich-Loeffler-Institute (NRL, FLI, Jena, Germany).

## Discussion

The microarray for *Salmonella* serogenotyping was validated against the gold standard and was evaluated as an economical, fast, accurate and easy-to-use diagnostic tool with a high potential for standardization and automated high throughput use. For identification of *Salmonella* using serogenotyping assays, several studies have already been published [13], [19], [20], [21]. The results of these publications showed high correlation of genotypic and phenotypic characterizations for genus *Salmonella*. Similar studies serogenotyping *Escherichia coli* [6], [30], [31] or *Chlamydia* [29], [32] also found a direct correlation of geno- and phenotype.

For *Salmonella,* at least four genes seem to be significant for specification of the genotype; *wzx* and *wzy* specify the O-serogroup, and the genes *fliC* and *fljB* specify the H antigens. To improve the correlation of geno-and phenotype, we analyzed fully sequenced *Salmonella* strains (Table 4) using theoretical hybridization with all probes on the microarray. The result was a similarity of over 99% between the phenotype represented by the antigenic formula and the genotype represented by the microarray based assay. Within the panel of theoretical reference experiments, strain *S.* ser. Typhimurium LT2 was the only one which was both fully sequenced and classically serotyped. Therefore, it was possible to compare the genotype represented through the NCBI database entry (NC_003197.1) with our theoretical experiments and subsequently with the real experiments using the same strain, *S*. ser. Typhimurium LT2. Theoretical and real experiments had a concordance of 100%. Even a deeper view of the signal-mismatch prediction from theoretical experiments resulted in a good correlation to the real experiment (Fig. 2). Only two probes showed signal strengths that differed from the results predicted by the theoretical experiment. Such discrepancies may occur due to secondary structure of the amplicon which decreases the binding strength to the probes. Finally, due to the high correlation between theoretical and real experiments, we conclude that the described serogenotyping assay will also correctly detect other *Salmonella* serovars.

A similar approach to the system described in this study was published by Franklin and colleagues in 2011 [13]. In this study, the labeling procedure is divided into two principal steps. The first step is a multiplex PCR targeting several regions of the *Salmonella* genome which are important for serogenotyping (i.e. *wzy*, *wzx*, *fliC*, *fljB*). In the second step, short SSELO primers (sequence-specific end labeling of oligonucleotides, [33]) bind specifically to the target regions in the multiplex PCR products and are elongated with single biotin-labeled dideoxynucleoside triphosphate molecules (biotin-ddCTP, biotin-ddGTP and biotin-ddUTP). The incorporation of these molecules also terminates any further elongation. During the microarray hybridization, the 3′ end labeled SSELO primers bind specifically to the complementary probes that are covalently attached on the array. Advantages of this method are the facility to recognize SNPs (single nucleotide polymorphism) and the potential to use DNA samples with low concentrations, i.e., DNA isolated directly from a field sample. However, a requirement for typing field samples is that the sample material contains only a single *Salmonella* serovar. For field samples containing more than one *Salmonella* strain, the serogenotype cannot be identified. Additionally, the labeling procedure is labor-intensive, complex and a target update would require, beside inclusion of new multiplex PCR primers, the introduction of new SSELO primer for detection on the microarray. With regard to the permanently increasing number of available sequences, the difficulties of up-dating and expanding this assay might pose a limitation for this concept.

A more recent method to identify *Salmonella* is a system using a microsphere-based liquid array [19], [20]. This method uses a set of beads which are coupled with probes for one attribute within the antigenic formula of *Salmonella* serovars. While the method is highly sensitive and specific, a multitude of different beads is required for every attribute within the antigenic formula (*e.g*., O-antigen). Therefore, at least three reactions have to be performed before obtaining the antigenic formula. A drawback of the method is the multiplex PCR used to amplify short DNA fragments which are then hybridized to the probes on the beads. Due to the inherent disadvantages of any multiplex PCR [34], [35], the options are limited for a further expansion of the assay beyond the serovars it currently recognizes.

The described microarray based serogenotyping assay for *Salmonella* overcomes most of these bottlenecks. It is easy-to-use, an unlimited expandability and fully automated data analysis, making it an attractive platform for a widespread application. The multiplex primer extension reaction used for labeling is highly specific, but exhibits low sensitivity, due to linear (non-exponential) amplification. However, for typing colony material of a fast growing organism, such as *Salmonella*, this is no issue. The use of colony material instead of original field samples allows both, to obtain the necessary amount of DNA and to ensure pureness and clonality of cultures to be genotyped. Besides, the limited amplification can prove to be an advantage under routine conditions as the assay becomes less susceptible to contamination. Using a classic multiplex PCR, the sensitivity is very high, but contaminants will also be amplified to a detectable level because of the near-exponential kinetics of a PCR. This fact might cause difficulties in high-throughput routine laboratories.

In our approach, primers and their respective probe binding sites are very close to each other. The probability of secondary structures (*e.g.*, hairpins) forming in short generated fragments is lower than in long fragments and this may increase signal intensity. Additionally, the use of single stranded DNA prevents the competition between probe and antisense strand and increases the probability of the single stranded amplicon binding to the probe. Labeling methods using biotin attached to primers were often used [36], but we assumed that, due to cross hybridizations of biotin labeled primer which are in relatively high concentrations, false positive signals will occur more often. In this study, biotin labeled dUTP was used for internal labeling of the multitude of single stranded amplicons. This method prevents false positive signals due to unused primer which bind on empty probes. Another significant advantage of the described serogenotyping method is the economical and ready-to-use availability of all components, even in large scales. For DNA isolation, we used standard DNA isolation kits from Roche or Qiagen. Furthermore, it is conceivable that, after heating at 100°C and RNase treatment (assay sensitivity may decreases due to single stranded RNA which may trap primer used in the multiplex linear DNA amplification), the crude cell extract could be used directly with this assay. All substances for the linear multiplex PCR and the labeling process are available as HybPlus Kit (Alere Technologies, Germany). Due to the standardized availability of all components, this method can immediately be used for routine serogenotyping of *Salmonella*. Up to 96 samples can be analyzed simultaneously.

So far, the serogenotyping assay shows the limitation of the inability to discriminate between serogroup A (O:2) and serogroup D1 (O:9). This is due to the high sequence similarity within the *rfb* region between strains of both serogroups. Within the genome of serogroup A strains, the *rfb* region has been shown to be a minor modification of a serogroup D1 *rfb* region; it has a frameshift mutation that inactivates *tyv*, a sugar biosynthesis gene required for the biosynthesis of tyvelose [37]. Serogroup D1 strains have tyvelose as their O-antigen side chain sugar, whereas serogroup A strains have paratose, the substrate for tyvelose, as its side chain sugar. Thus, a small genetic change is responsible for a substantial O-antigen difference. Additional probes, including *lygA*, *lygD*, *sefA*, *sefB* and *sefC*, which were only described for serovar Enteritidis [38], [39], also give positives signals for serovar Nitra. Additionally, *S.* ser. Blegdam (O9:g,m,q:-) showed an identical pattern on the microarray, but in this case the antigenic formula is highly similar to *S.* ser. Enteritidis (O9:g,m:-). This result showed how closely related these serovars are to each other. A similar observation between the serogroups A and D1 were made for the serovars Dublin (O9:g,p:-) and Kiel (O2:g,p:-), where additional probes for *SeD_A1100*, *SeD_A1101 and SeD_A1102* were also positive with serovar Kiel. This observation may indicate a high degree of relationship between these two serovars. Furthermore, we assume a high genome sequence similarity between Panama (O9:l,v:1,5) and Koessen (O2:l,v:1,5) as the microarray pattern were also identical. Paratyphi A could be unambiguously identified due to the probes of intergenic region SSPAI. With the knowledge about the genotype of these described serovars a question arises: Is there a need to differentiate between serogroup D1 and A or between g,m and g,q/g,m,q (both a mutation variant of g,m)? Or should the Kauffmann-White Scheme be updated based on our knowledge of *Salmonella* genomics and the current role of serotyping (*e.g.*, a first line typing method prior to modern molecular methods). Nevertheless, a future version of the assay aims to include probes to discriminate such important zoonotic pathogens as *S.* ser. Enteritidis and *S.* ser. Dublin. For this propose, genome databases (*e.g.*, NCBI) are regularly screened for new *Salmonella* sequences.

In addition, all isolates (n = 34) in which resistance genes were found, were also tested phenotypically. A clear correlation between AMR genotype detected by the *Salmonella* array and the AMR phenotype was observed (Table 5, Table S2) for most genes. However, the genes *sul1* and *sul2* are known to mediate resistance to sulfamethoxazol, but in the study only co-trimoxazol, a mixture comprising trimethoprim and sulfamethoxazol, was tested. Strains that harbored only *sul* genes were found to be susceptible when being tested with co-trimoxacol. Resistance to co-trimoxazol was associated to the presence of a *sul* gene (*sul1*, *sul2* or *sul3*) combined with a *dfr* gene (*dfrA1*, *dfrA12*, *dfrA13*, *dfrA14*, *dfrA15* or *dfrV*). Similar correlations between AMR pheno- and genotype were found in other studies using microarrays for *E. coli* and *Salmonella* [40], [41]. In these studies, all probes and primers used in this study were already evaluated by testing phenotypic antimicrobial resistance in parallel.

Another interesting result was the detection of an extended-spectrum beta-lactamase (ESBL) gene, *ctxM1,* in *S.* ser. Anatum strain AMR07 (Table S2). This gene has previously been described several times for *Salmonella* species [42], [43]. In one isolate, ampicillin resistance and a positive nitrocephin test were observed despite a negative genotyping result. For this observation, two explanations are possible: (a) the probe/primer combination of our assay did not bind on the known beta-lactamase gene due to random mutation(s) within the binding side of either the primer or the probe, or (b) the isolate carried a beta-lactamase gene which was not yet described for *Salmonella* and which therefore was not included into the *Salmonella* assay. Regarding the unexplained streptomycin resistances, it might be speculated that aminoglycoside modifying enzymes for which the corresponding genes were not covered by the array or efflux systems as known from other bacteria [44] might have played a role.

The study, with a panel of 105 different *Salmonella* isolates, demonstrates the high correlation between genotype classification by the serogenotyping assay and phenotype representation by the classical serotyping method. Approximately 90% of the strains were correctly identified (Table 6). The accuracy depends strongly on the underlying database used by the PatternMatch software, but any unknown serovar encountered can easily be included in the database and software upon conventional identification. The main drawback of the assay turned out to be the similarity of patterns between different *Salmonella* serovars, as described above. With the primer-probe design used here, we were not able to discriminate between these problematic strains, mainly because of the high sequence similarity in the target genes of serogroup A and D1. However, all these ambiguous strains are very rare in a clinical environment, each being reported less than 10 times worldwide during the last 10 years ([1], www.cdc.gov/ncezid/dfwed/PDFs/SalmonellaAnnualSummaryTables2009.pdf), and additional probes can easily be introduced should a need arise, or should new sequence information become available.

Due to the absence of a probe which can determine the genetic loci of the O:5 epitope, the isolate *S.* ser. Typhimurium var. Copenhagen, which is O:5-negative by serotyping, was identified as *S*. ser. Typhimurium; this minor mistake was regarded as a correct hit.

Another minor limitation is that R- forms (rough forms) cannot be identified using the current array as observed in one isolate of Infantis. R- forms mainly result from mutations of genes within the lipopolysaccharides core [45]. Mutations within the genes *rfa* (glycosyltransferase), *galE* (UDP-galactose epimerase), or *galF* (UDP-glucose pyrophosphorylase) can cause an interruption of the biosynthesis of the lipopolysaccharides. No probes detecting such mutations were included to the array, and failure to identify R- forms regarded as minor issue.

The described assay for serogenotyping is the basis for a fast method to identify *Salmonella* serovars. We believe that the usage of this assay in a routine laboratory setting is warranted due to the high correlation between serotype and genotype. An advantage of the genotype as the basis for serovar identification is that phenotypic differences (*e.g*., R-forms that are difficult to analyze by classical serology) play no role. Furthermore, the serogenotyping assay could be used worldwide, where antisera are not available. In such areas, a *Salmonella* infection in livestock or *Salmonella* contamination in food could be identified very quickly. *Salmonella* outbreaks could consequently be retraced to their origin. This microarray based assay is a powerful tool for epidemiological studies, as many samples can be analyzed rapidly and in parallel. For such cases, a point-of-care application represents an ideal standard. During an outbreak situation, this assay could be extremely helpful to identify the outbreak isolate including AMR genotype within hours after they are obtained as clonal serovar. Finally, an interlaboratory comparison in cooperation with several international reference centers will follow in the near future.

## Supporting Information

Table S1 **Probe-matching matrix used to construct the theoretical hybridization pattern of the fully sequenced strains listed in NCBI database.**
(XLSX)

Table S2 **Comparison of the AMR-genotype and AMR-phenotype of 34** ***Salmonella*** **strains.**
(XLSX)
